# A novel sucrose-inducible expression system and its application for production of biomass-degrading enzymes in *Aspergillus niger*

**DOI:** 10.1186/s13068-023-02274-7

**Published:** 2023-02-13

**Authors:** Lu Wang, Yijia Xie, Jingjing Chang, Juan Wang, Hong Liu, Mei Shi, Yaohua Zhong

**Affiliations:** 1 State Key Laboratory of Microbial Technology, Institute of Microbial Technology, Shandong University, Qingdao, 266237 People’s Republic of China; 2 Qingdao Academy, Qingdao, 266111 People’s Republic of China

**Keywords:** *Aspergillus niger* ATCC 20611, β-Fructofuranosidase, P*fopA*, Sucrose-inducible, β-glucosidase, Chitinase, N-Acetylglucosamine, Biomass

## Abstract

**Background:**

Filamentous fungi are extensively exploited as important enzyme producers due to the superior secretory capability. However, the complexity of their secretomes greatly impairs the titer and purity of heterologous enzymes. Meanwhile, high-efficient evaluation and production of bulk enzymes, such as biomass-degrading enzymes, necessitate constructing powerful expression systems for bio-refinery applications.

**Results:**

A novel sucrose-inducible expression system based on the host strain *Aspergillus niger* ATCC 20611 and the β-fructofuranosidase promoter (P*fopA*) was constructed. *A. niger* ATCC 20611 preferentially utilized sucrose for rapid growth and β-fructofuranosidase production. Its secretory background was relatively clean because β-fructofuranosidase, the key enzyme responsible for sucrose utilization, was essentially not secreted into the medium and the extracellular protease activity was low. Furthermore, the P*fopA* promoter showed a sucrose concentration-dependent induction pattern and was not subject to glucose repression. Moreover, the strength of P*fopA* was 7.68-fold higher than that of the commonly used glyceraldehyde-3-phosphate dehydrogenase promoter (P*gpdA*) with enhanced green fluorescence protein (EGFP) as a reporter. Thus, *A. niger* ATCC 20611 coupled with the P*fopA* promoter was used as an expression system to express a β-glucosidase gene (*bgla*) from *A. niger* C112, allowing the production of β-glucosidase at a titer of 17.84 U/mL. The crude β-glucosidase preparation could remarkably improve glucose yield in the saccharification of pretreated corncob residues when added to the cellulase mixture of *Trichoderma reesei* QM9414. The efficacy of this expression system was further demonstrated by co-expressing the *T. reesei*-derived chitinase Chi46 and β-N-acetylglucosaminidase Nag1 to obtain an efficient chitin-degrading enzyme cocktail, which could achieve the production of N-acetyl-D-glucosamine from colloidal chitin with a conversion ratio of 91.83%. Besides, the purity of the above-secreted biomass-degrading enzymes in the crude culture supernatant was over 86%.

**Conclusions:**

This P*fopA*-driven expression system expands the genetic toolbox of *A. niger* and broadens the application field of the traditional fructo-oligosaccharides-producing strain *A. niger* ATCC 20611, advancing it to become a high-performing enzyme-producing cell factory. In particular, the sucrose-inducible expression system possessed the capacity to produce biomass-degrading enzymes at a high level and evade endogenous protein interference, providing a potential purification-free enzyme production platform for bio-refinery applications.

**Supplementary Information:**

The online version contains supplementary material available at 10.1186/s13068-023-02274-7.

## Background

Filamentous fungi possess an impressive natural capacity to secrete copious amounts of proteins (mainly hydrolytic enzymes) into the environment, highlighting their intriguing roles as hosts for the industrial-scale production of homologous and heterologous enzymes [1, 2]. In particular, *Aspergillus niger* has gained immense attention due to its GRAS (generally regarded as safe) status as a preferred, versatile cell factory [2, 3]. Proteome analysis reveals that *A. niger* can secrete a diverse range of glycoside hydrolases to cope with changing carbon sources, such as glucoamylase in the maltose-grown culture, xylanase in the xylose-grown culture [4, 5]. This property not only makes *A. niger* a great reservoir of glucoamylase and xylanase but also provides robust and adjustable promoters of glycoside hydrolases for enzyme production [2, 6]. For example, the promoter of the highly expressed glucoamylase-encoding gene (P*glaA*) has been one of the foremost inducible promoters for driving the expression of various heterologous enzymes, including β-glucosidase and cellobiohydrolase [3, 7, 8]. However, certain highly secreted background proteins, such as alpha-amylases and alpha-glucosidases, are also expressed with target enzyme under inducing conditions, which could restrict the ascension of target enzyme production [9–11]. Meanwhile, complex protein background is highly disadvantageous for the production of a specific protein or a relatively pure monocomponent enzyme, as it renders the purification of the target enzyme more tedious and costly [9, 12].

Attempts have been made to express enzymes in the *A. niger* host strains with low protein backgrounds [9, 13–15]. The tannase from *A. niger* and the Nuclease P1 from *Penicillium citrinum* have been successfully expressed in a low-background host strain *A. niger* Bdel4, which was achieved by deleting the major secreted proteins: glucoamylase, acid α-amylase and two putative α-amylases (An05g02100 and An12g06930) [13, 14]. Alternatively, Dong et al. [15] knocked in two copies of trehalase genes from *Myceliophthora thermophila* at the loci of two highly expressed extracellular secreted alpha-amylases by the CRISPR/Cas9 tool, resulting in a 793.60% increase in trehalase activity along with a substantial abatement of protein background. Nonetheless, there is a high burden associated with achieving low secretion background hosts by multiple gene deletions, which necessitates the use of multiple screening markers in a single host and efficient gene targeting system [2, 12]. Considering that the expression of amylolytic enzymes is controlled by the transcription activator AmyR, Zhang et al. [9] constructed an host strain ∆*amyR* with a low background of protein secretion by deleting *amyR* in *A. niger* CICC246. Whereas, downstream promoters of AmyR, such as P*glaA*, P*amyA*, and P*amyB*, reduced their activity in this strain. In this case, constitutive promoters, such as the commonly used glyceraldehyde-3-phosphate dehydrogenase promoter (P*gpdA*), have the advantage of independent AmyR regulation and can be candidates for expressing the target enzymes. Zhang et al. [16] adopted a strategy of increasing the copy numbers of *gpd* box to enhance the transcription efficiency of the P*gpdA* promoter. However, its transcription efficiency was still limited, which was only 65% of that of P*glaA* for *A. niger* CICC2462. Therefore, the establishment of a novel *A. niger* expression system with high efficiency and low endogenous protein interference is essential for expressing target enzymes and scaling up enzyme production to the industrial level.

Biomass, such as cellulose and chitin, is the most abundant renewable resource in nature and has been devoted to its valorization as a viable alternative to petroleum-based fuels, chemicals, and materials [17–19]. Nevertheless, enzymatic degradation of biomass into monomers that can be subsequently utilized by the fuel or chemical industries still presents significant barriers, such as low efficiency and high costs of biomass-degrading enzymes [18, 19]. Thus far, the commercial cellulases for cellulose degradation are predominantly from filamentous fungi, of which, *Trichoderma reesei* is extensively used as a source of cellulases but has a less abundance of β-glucosidase (BGL) [18, 20]. BGL plays a vital role in lignocellulose degradation by undertaking the rate-limiting final step of hydrolyzing cellobiose, which is an intermediate product and also an inhibitor of cellulase activities, into glucose [21, 22]. As such, supplementing the *T. reesei* cellulase preparation with exogenous BGLs is of crucial importance for efficient biomass degradation [20, 23, 24]. The addition of purified BGL to the *T. reesei* Rut-C30 cellulase preparation has been proven to bring about an 80% increase in glucose yield during the saccharification of pretreated cornstalk [23]. In addition, Xia et al. [24] applied an engineered BGL from *Talaromyces leycettanus* JCM12802 that collaborated with the commercial *T. reesei* cellulase Celluclast 1.5L, providing a two-fold improvement in saccharification efficiency. However, the preparation of purified BGL also introduced additional complexity and cost, making it necessary to find a suitable fungal expression system for the production of high-purity BGL [22]. Unlike the enzymatic degradation of cellulose is widely used in industry, the enzymatic degradation of chitin cannot be scaled up because the monomeric subunit N-acetyl-D-glucosamine (GlcNAc) conversion rate is always low [25, 26]. Moreover, the purity of GlcNAc remains challenging [27]. Most crude chitinolytic enzymes from natural microorganisms always produce a mixture of GlcNAc and N-acetyl chitooligosaccharides from the enzymatic hydrolysis of chitin [28–30]. In general, the degradation of chitin into GlcNAc involves the synergistic action of chitinase and β-N-acetylglucosaminidase (GlcNAcase) [31]. Thus, heterologous expression, purification, and assembly of chitinase and GlcNAcase in appropriate proportions are currently common strategies to improve the hydrolysis efficiency of colloidal chitin [31–33]. For example, under the co-action of purified chitinase SaChiA4 and GlcNAcase SvNag2557 with a mass ratio of 1:2, the final conversion rates of colloidal chitin to GlcNAc reached 80.2% [31]. However, the bioprocess for GlcNAc production still needs to be optimized since chitin-degrading enzyme use for industrial applications is at an unreasonable cost [25, 26]. In addition, although *Escherichia coli* is the frequently used heterologous system for the expression of chitinolytic enzymes, the expression of fungal chitinases in a bacterial system is restricted by post-translational modifications, and thus filamentous fungi systems can be better alternatives [1, 27, 31, 32]. In this regard, the cost-effective and highly efficient production of biomass-degrading enzymes, such as BGL and chitinolytic enzymes, in low-background *A. niger* strains, would make an immense contribution to increasing the economic feasibility of large-scale biomass-based bio-refineries.

β-fructofuranosidase (FopA) is the key biocatalyst for commercial sucrose-to-fructooligosaccharides (FOS) biotransformation [34]. Due to the high FopA productivity, *A. niger* ATCC 20611 has long been used for FOS production in the food industry [34–36]. Despite its importance as an industrially relevant organism, strain improvement by genetic engineering is limited until recently, a PEG-mediated transformation method was successfully developed in *A. niger* ATCC 20611 [36, 37]. Given that *A. niger* ATCC 20611 can use sucrose as a fermentation carbon source to produce high-level FopA, the promoter of the *fopA* gene (P*fopA*) could be a promising candidate for controlling gene expression in *A. niger* ATCC 20611. Moreover, FopA appears to accumulate mainly in the mycelium cells, since the mycelium cells are always utilized for the sucrose-to-FOS biotransformation [35–37]. Thus, the extracellular protein background of *A. niger* ATCC 20611 may be relatively low compared to the general enzyme-producing *A. niger* host strains, such as *A. niger* CICC2462 and CBS 513.88, which preferentially use starch for growth and enzyme production [6, 9]. Therefore, *A. niger* ATCC 20611 was hypothesized to be an outstanding host strain for the construction of a sucrose-inducible expression platform. To validate the hypothesis, the distribution of FopA and the extracellular protease activity of this strain under sucrose conditions were explored. In parallel, the induction pattern of P*fopA* was characterized, and the strength of P*fopA* was assessed using EGFP as a reporter. On this basis, an *A. niger* sucrose-inducible expression system was constructed. The system allowed high titer production of a β-glucosidase (BGLA) from *A. niger* C112 and a chitinolytic enzyme cocktail (including the chitinases Chi46 and GlcNAcase Nag1) from *Trichoderma reesei*. More importantly, these recombinant enzymes were enabled to be purification-free and used directly to degrade biomass materials.

## Results

### Effect of carbon sources on the mycelial growth and β-fructofuranosidase production

**Fig. 1:**
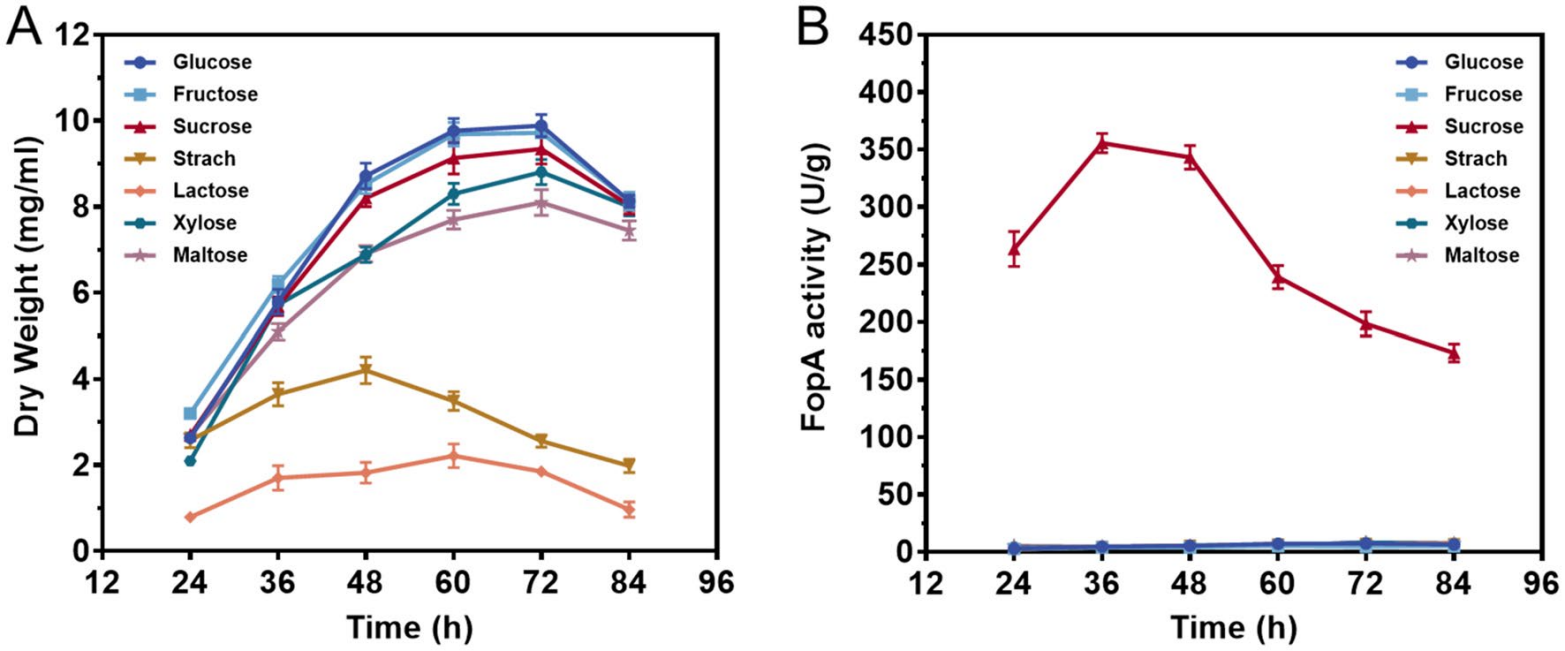
Effect of carbon source on mycelial growth (**A**) and β-fructofuranosidase (FopA) production (**B**) of *A. niger* ATCC 20611. *A. niger* ATCC 20611 was cultured in the fermentation medium with 2.0% fructose, glucose, sucrose, starch, lactose, xylose, or maltose as carbon sources. Values are the average of three replicates and error bars are the standard deviation (SD) of these three replicates

*A. niger* ATCC 20611 was inoculated in FM with different carbon sources for 84 h to assess its growth pattern, nutritional preference, and FopA production. Figure 1 depicted the time profiles of mycelial growth and FopA production during shake flask fermentation. As shown in Fig. 1A, when fructose, glucose, sucrose, xylose, or maltose were used as the carbon source, mycelia showed excellent growth, and biomass accumulated rapidly. Moreover, the highest biomass concentration (approximately 10.14 mg/mL) was obtained in glucose and fructose after 72 h of cultivation, followed by sucrose (9.42 mg/mL) at 72 h. While growth in starch or lactose was relatively poor, especially the latter exhibiting the lowest growth of about 2.16 mg/mL. Meanwhile, the fungal cultures from different carbon sources were collected for the FopA activity assay (Fig. 1B). It was found that only the sucrose-grown culture possessed high FopA activity, especially, the maximum production of FopA reached 355.67 U/g at 36 h during the exponential growth stage. As for the other carbon source-grown cultures, the enzyme activity was below 10 U/g (Fig. 1B). Thus, sucrose was the preferred carbon source for *A. niger* ATCC 20611 growth as well as an essential inducer for the β-fructofuranosidase production.

### Low background of hydrolase secretion and low level of extracellular protease production in *A. niger* ATCC 20611

**Fig. 2:**
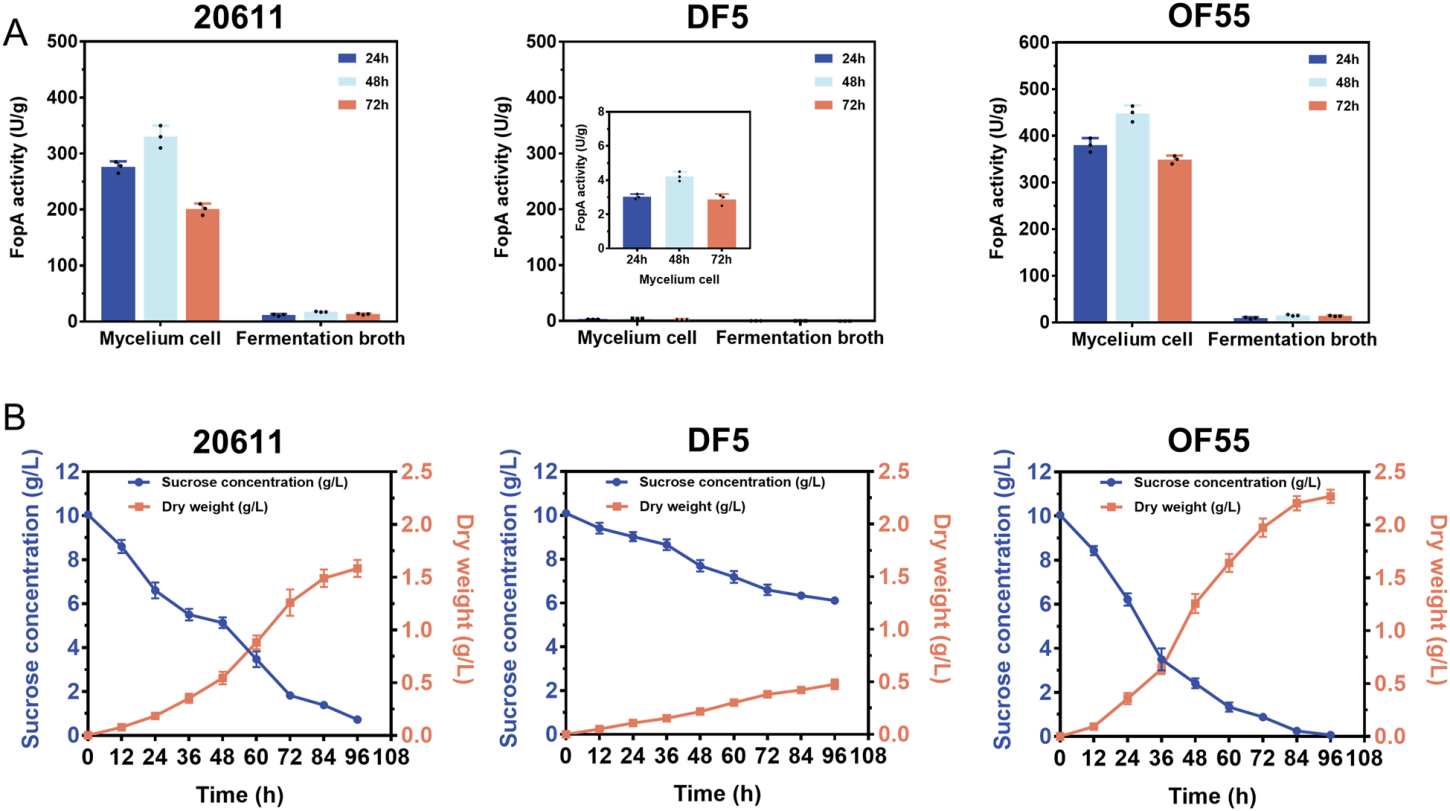
Effect of deletion or overexpression of *fopA* on the β-fructofuranosidase (FopA) production and sucrose utilization capacity of *A. niger* strains. **A** Distribution of FopA activity of the ATCC 20611, *fopA* deletion strain DF5, or *fopA* overexpression strain OF55 cultured in fermentation medium with 2% sucrose as the carbon source. **B** Biomass accumulation and sucrose consumption of ATCC 20611, DF5, or OF55 cultured in CD medium with 1% sucrose as the carbon source. Values are the average of three replicates and error bars are the standard deviation (SD) of these three replicates

Although FopA from *A. niger* ATCC 20611 has been characterized and used as a key biocatalyst in the industrial sucrose-to-FOS biotransformation for more than 30 years [38], little is known about the role that FopA plays in the sucrose utilization of this strain. To determine whether FopA was the key enzyme responsible for mycelial growth under sucrose conditions, the FopA-encoding gene (*fopA*) was deleted and overexpressed in *A. niger* ATCC 20611 to construct DF5 and OF55 strains, respectively. Then, the fungal growth and sugar consumption of the resultant mutants in CD media containing sucrose as the sole carbon source were measured. It was found that disrupting *fopA* resulted in a complete loss of the FopA activity and was accompanied by a severe defect in mycelial growth (Fig. 2A, B). In detail, at the end of the culture period, the *fopA* deletion strain DF5 displayed only 26.67% of biomass accumulation with respect to the parental strain (Fig. 2B). Correspondingly, the sucrose consumption of DF5 was 67.32% less than that of *A. niger* ATCC 20611 (Fig. 2B). Instead, compared to the parental strain *A. niger* ATCC 20611, the *fopA* overexpression strain OF55 showed a 1.31-fold increase in FopA production, a 48.26% increase in biomass accumulation, and a much shorter time required to consume sucrose (Fig. 2A, B). These data revealed the crucial role of FopA in the sucrose utilization of mycelia. Subsequently, the distribution of FopA activity in mycelium cells and the fermentation broth was investigated. A significant accumulation (approximately 90.23%) of the FopA activity in the mycelial cells was observed in the sucrose-grown culture, indicating *A. niger* ATCC 20611 was a promising host strain with a low background of hydrolase secretion (Fig. 2A).

Considering that proteolytic degradation by fungal proteases is one of the major bottlenecks interfering with efficient enzyme production [39, 40], extracellular protease produced by *A. niger* ATCC 20611 under the sucrose condition was determined. Equal amounts of conidia of *A. niger* MGG029, which is deficient in the expression of several protease genes due to a regulatory mutation, and *A. niger* ATCC 20611, were inoculated on the skim milk-agar plates for 3 d cultivation. The ratio of halo diameter to colony diameter was calculated to imply protease secretion ability. As depicted in Additional file 1: Figs. S1A, B, a clear halo was formed around the colony of MGG029 and the ratio of the halo diameter to the colony diameter reached 1.50, while the proteolytic halo around ATCC 20611 was invisible. In addition, the proteolytic activities toward azocasein in the fermentation broth supernatants of the fungal strains were given in Additional file 1: Fig. S1C. The extracellular protease activity of ATCC 20611 was significantly lower, only 16.59% of that of MGG029. Taken together, these findings suggest that *A. niger* ATCC 20611 had the advantage of low endogenous protein interference and was suitable to be developed as a host for enzyme production.

### Effect of sucrose concentrations on the β-fructofuranosidase production

**Fig. 3:**
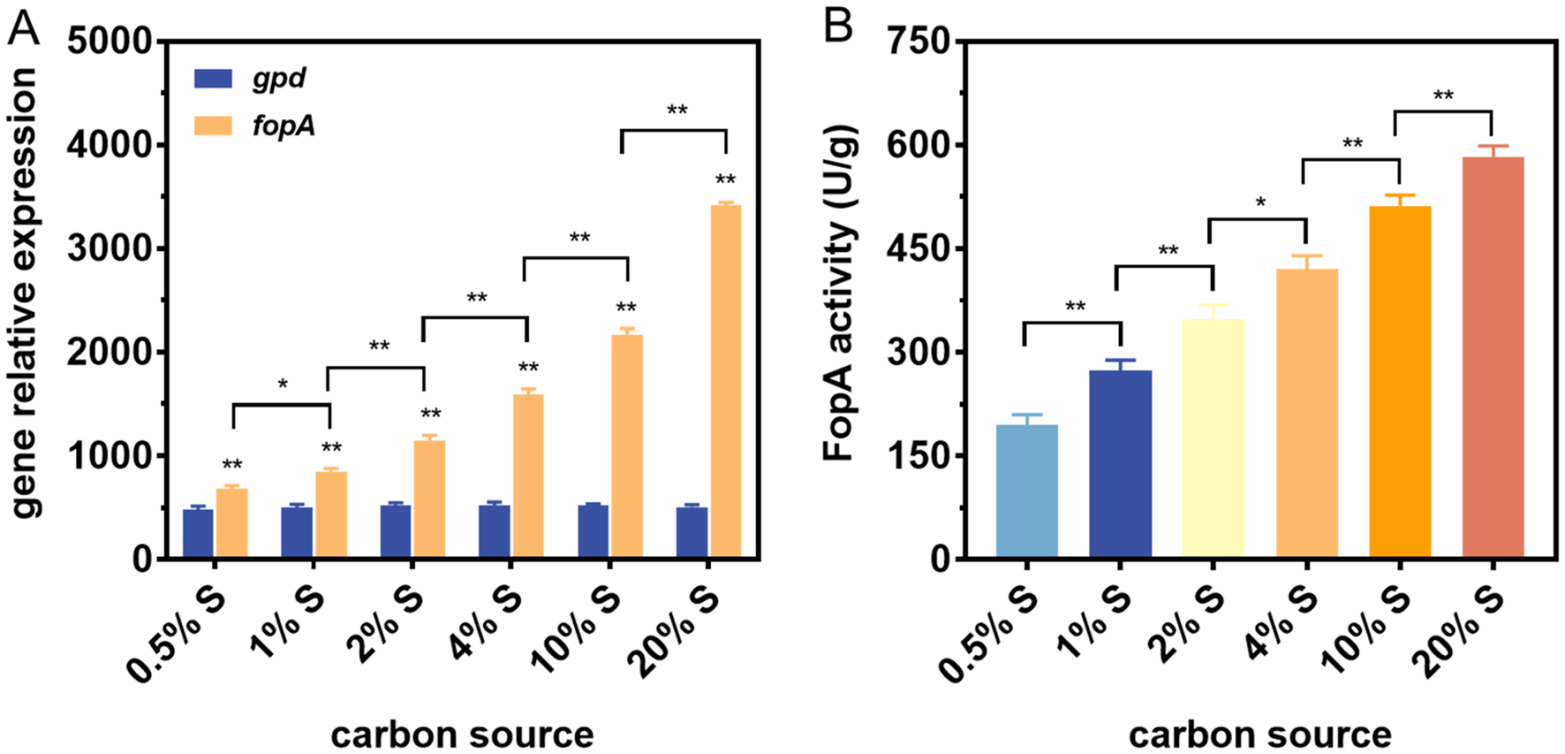
Effect of different sucrose concentrations on *fopA* expression and β-fructofuranosidase (FopA) production in *A. niger* ATCC 20611. **A** RT-qPCR analysis of the changes of *fopA* expression levels in response to various sucrose concentrations. *Actin* was used as the reference gene and *gpd* was used as the control gene. The 2^−ΔΔCt^ method was used for calculating relative expression levels. **B** The changes of β-fructofuranosidase production levels in response to different sucrose concentrations. Values and standard deviation (SD) of triplicates are presented in the figure. Asterisks indicate statistically significant differences (*p < 0.05, **p < 0.01) as assessed by Student’s t test

As mentioned above, FopA was only expressed in the presence of sucrose, implying that the promoter of the *fopA* gene (P*fopA*) was a sucrose-inducible promoter. To further characterize the induction pattern of P*fopA*, reverse transcription quantitative real-time PCR (RT-qPCR) was performed to monitor the changes in the *fopA* gene transcript levels in response to different sucrose concentrations (Fig. 3A). *fopA* transcript levels were enhanced with increasing sucrose concentrations ranging from 0.5 to 20%. For instance, *fopA* transcript levels were 5.11-fold higher at 20% (w/v) sucrose concentration than at 0.5% (w/v) sucrose concentration. Moreover, the strength of P*fopA* was compared to the promoter of the key glycolytic enzyme glyceraldehyde-3-phosphate dehydrogenase gene (P*gpd*). It was found that the *gpd* gene was constitutively transcribed in the strain, regardless of the initial sucrose concentration used (Fig. 3A). Moreover, significantly higher transcript levels were observed for *fopA* than for *gpd* under all cultivation conditions (Fig. 3A). Even at the minimal sucrose induction concentration (0.5%), *fopA* transcript level was up to 1.43-fold higher than that of *gpd*, suggesting that P*fopA* was a strong promoter. To evaluate whether the increase in the *fopA* transcript level led to enhanced enzyme production, the FopA activities of mycelial cells grown in FM containing different sucrose concentrations were measured. As depicted in Fig. 3B, the FopA production showed a positive correlation with the *fopA* transcript levels at the sucrose concentrations used. Moreover, the maximum FopA activity could reach up to 583.23 U/g at a 20% sucrose concentration, which was 3.05-fold and 1.47-fold higher than the FopA activity of mycelial cells grown in FM with 0.5% and 4.0% sucrose as the carbon source, respectively. These data indicated that the sucrose concentration had a significant impact on the ability of P*fopA* to drive gene expression. However, it should be noted that high sucrose concentrations resulted in increased enzyme production but impaired mycelial cell growth. For example, when 20% (w/v) sucrose was used as a carbon source, there was a 64.45% reduction in biomass accumulation compared to 4% sucrose (data not shown). Thus, considering the optimal balance of FopA production and mycelial cell viability, an initial sucrose concentration of 4% was chosen for subsequent strain fermentation.

**Fig. 4:**
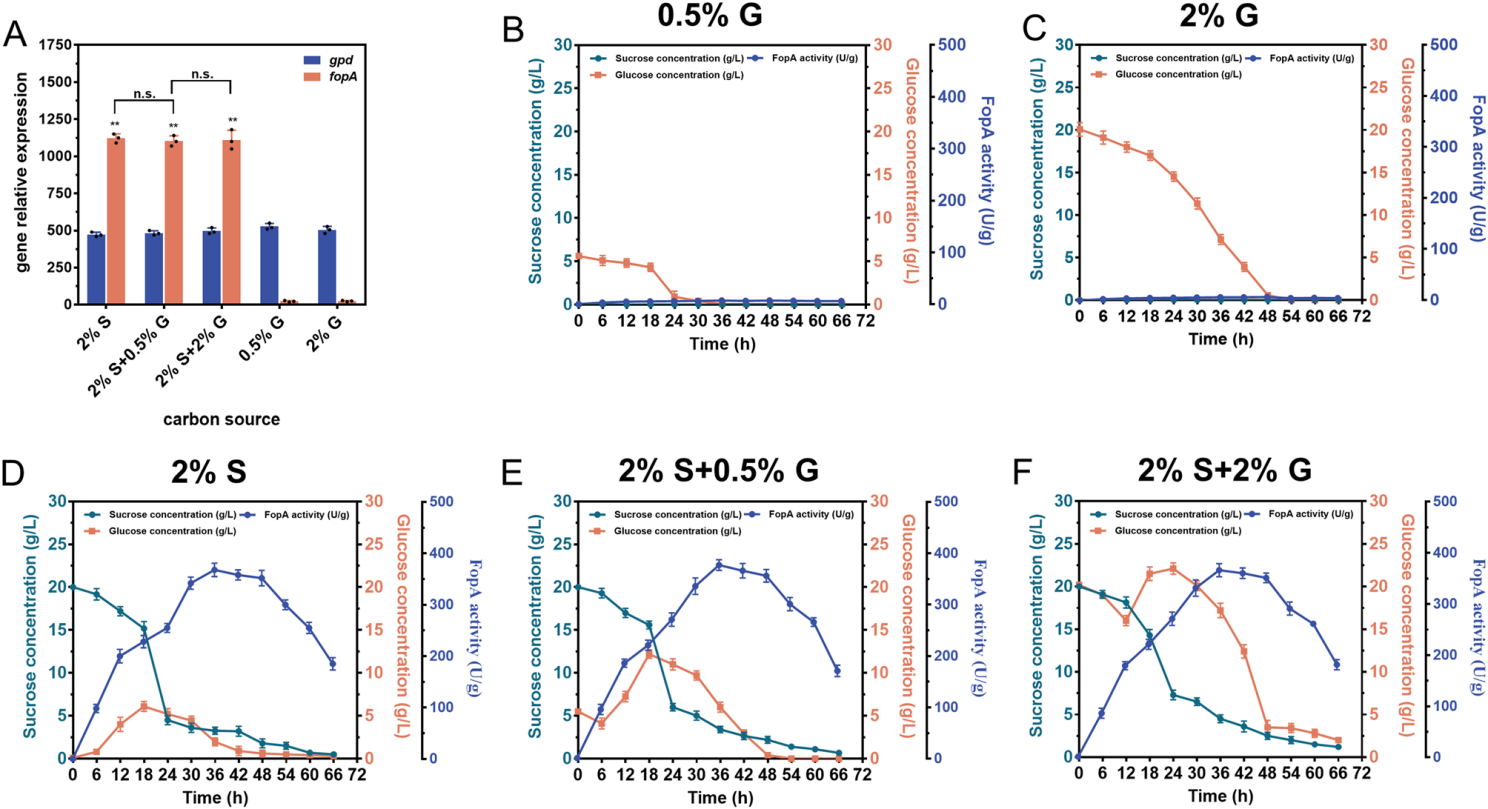
Effect of the presence of glucose on *fopA* expression and β-fructofuranosidase (FopA) production in *A. niger* ATCC 20611. **A** RT-qPCR analysis of the changes of *fopA* expression levels in response to the presence of glucose. *Actin* was used as the reference gene and *gpd* was used as the control gene. The 2^−ΔΔCt^ method was used for calculating relative expression levels. Sugar consumption and FopA production of ATCC 20611 cultured in the fermentation medium with 0.5% glucose (**B**), 2% glucose (**C**), 2% sucrose (**D**), 2% sucrose plus 0.5% glucose (**E**), 2% sucrose plus 2% glucose (**F**) as the carbon source. Values and standard deviation (SD) of triplicates are presented in the figure. Asterisks indicate statistically significant differences (**p < 0.01) as assessed by Student’s t test. n. s., no significant differences

Furthermore, *A. niger* ATCC 20611 was inoculated into FM containing sucrose (S) and glucose (G) as the mixed carbon sources (2% S + 0.5% G, or 2% S + 2% G) to test whether glucose had a negative impact on sucrose induction. As shown in Fig. 4A, the *gpd* gene was constitutively transcribed in the strain regardless of the carbon source used. *fopA* was not transcribed when glucose was used as the carbon source but was excessively transcribed when sucrose was present, and the transcription level of *fopA* of cultures with both sucrose and glucose was similar to that of cultures with sucrose alone (Fig. 4A). The FopA production and sugar consumption of this strain under different culture conditions were further explored. When glucose was used as the sole carbon source (0.5–2% G), glucose was rapidly consumed but no FopA was produced (Fig. 4B, C). When different concentrations of glucose (0.5–2%) were added to 2% sucrose, the strain still could utilize sucrose in the early stages of fermentation (0–18 h) simultaneously with the rapid accumulation of FopA. Moreover, no reduction in FopA activity was detected during the fermentation process compared to ATCC 20611 grown with sucrose as a single carbon source (Figs. 4D–F). These results indicated that P*fopA* was not regulated by glucose repression, so it was able to mediate the fast production of enzymes after sucrose induction, which could facilitate a highly efficient fermentation process with complex carbon sources for enzyme production.

### Comparison of the sucrose-inducible promoter P*fopA* and the commonly used constitutive promoter P*gpdA* from *A. nidulans* for enhanced green fluorescent protein (EGFP) expression

**Fig. 5:**
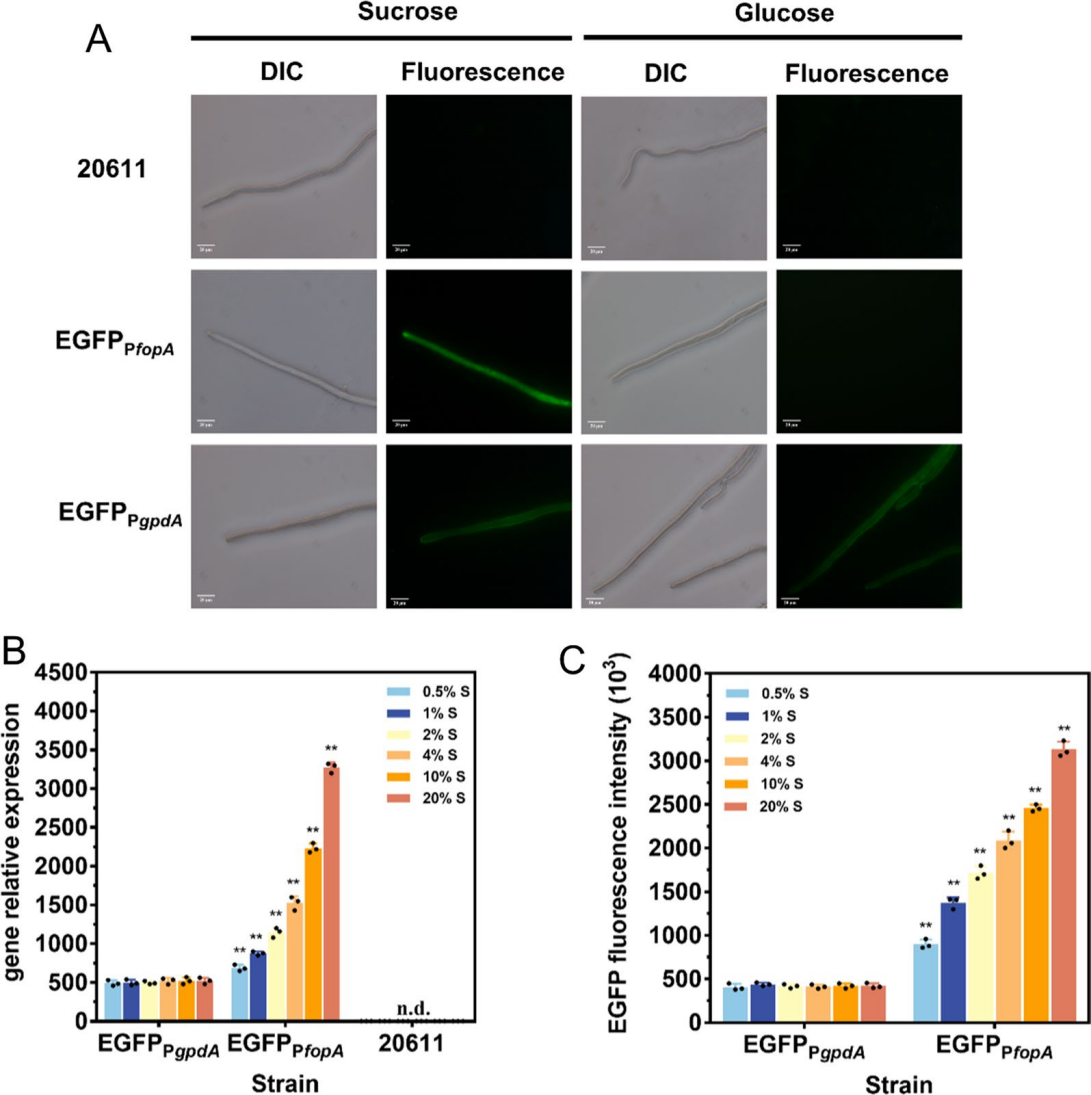
Comparison of P*fopA* and the constitutive promoter P*gpdA* from *A. nidulans* for enhanced green fluorescent protein (EGFP) expression in *A. niger*. **A** Observation of EGFP fluorescence of EGFPP*fopA* and EGFPP*gpdA* grew on the MM-sucrose and MM-glucose plates. Images in DIC and fluorescence channels were acquired at 40 × magnification and 600 ms exposure using a Nikon positive fluorescence microscope. The scale bar is 20 μm. **B** RT-qPCR monitoring of *egfp* expression levels under the control of P*fopA* and P*gpdA* at different sucrose concentrations. *Actin* was used as the reference gene. The 2^−ΔΔCt^ method was used for calculating relative expression levels. **C** Quantitative analysis of EGFP fluorescence intensity of EGFPP*fopA* and EGFPP*gpdA* at different sucrose concentrations. Values and standard deviation (SD) of triplicates are presented in the figure. Asterisks indicate statistically significant differences (**p < 0.01) as assessed by Student’s t test. n.d., not detected

The P*gpdA* promoter from *A. nidulan* is the most frequently used promoter for the genetic engineering of *Aspergillus* spp. to provide high levels of constitutive expression of target genes [41]. Moreover, P*gpdA* has been successfully used to drive the expression of β-fructofuranosidase variants in *A. niger* ATCC 20611 [36, 37]. Here, the strength of P*fopA* was compared with that of P*gpdA* using enhanced green fluorescent protein (EGFP) as a reporter. The EGFP expression cassettes under the control of P*fopA* and P*gpdA* were constructed to generate *A. niger* strains, EGFP_P*fopA*,_ and EGFP_P*gpdA*_, respectively. The conidia of EGFP_P*fopA*_, EGFP_P*gpdA*_, and the parental strain ATCC 20611 were spotted on MM-sucrose and MM-glucose plates with coverslips and grown at 30 °C for 48 h. The mycelia attached to the coverslips were analyzed by fluorescence microscopy under blue light (Fig. 5A). The mycelia of ATCC 20611 did not show fluorescence on sucrose or glucose. While the strain EGFP_P*gpdA*_ displayed strong cytoplasmic EGFP fluorescence on both sucrose and glucose due to the constitutive expression of EGFP. As for EGFP_P*fopA*_, a high intensity of fluorescence emission in the mycelia was observed under the sucrose condition, while no fluorescence was shown under the glucose condition. These findings confirmed that P*fopA* and P*gpdA* successfully drove *egfp* expression, additionally, the expression of the *egfp* gene under the control of P*fopA* was completely repressed in the absence of sucrose.

The strains were subsequently subjected to flask-level cultivation under different sucrose concentration conditions (0.5, 1, 2, 4, 10, and 20%). The transcription levels of the *egfp* gene driven by P*gpdA* and P*fopA* were quantified by RT-qPCR (Fig. 5B). The *egfp* gene was constitutively transcribed in the strain EGFP_P*gpdA*_ regardless of the sucrose concentration, while the transcription levels of *egfp* in the strain EGFP_P*fopA*_ were 1.44 to 6.32 times higher than those of the strain EGFP_P*gpdA*_ after 0.5 to 20% sucrose induction. In agreement with this observation, the intensity of fluorescence of EGFP_P*gpdA*_ on sucrose did not vary according to the concentrations tested (0.5, 1, 2, 4, 10, and 20%). The fluorescence intensity of EGFP_P*fopA*_ was dependent on the used sucrose concentration, and the value was gradually elevated in response to 0.5 to 20% sucrose (Fig. 5C). As a result, the fluorescence intensity of EGFP_P*fopA*_ could reach 7.68-fold higher than that of EGFP_P*gpdA*_ at 20% (w/v) sucrose concentration. Even at the minimum induced concentration, the fluorescence intensity of EGFP_P*fopA*_ was still 2.25-fold enhanced than that of EGFP_P*gpdA*_. Thus, P*fopA*-driven *egfp* expression was obviously dependent on sucrose concentration, and the transcriptional activation capacity of P*fopA* was significantly higher than that of P*gpdA*. These results indicated that the sucrose-inducible promoter P*fopA* appeared to be a powerful promoter to express heterologous enzymes in *A. niger* ATCC 20611.

### Sucrose-inducible P*fopA*-driven expression of β-glucosidase in *A. niger* ATCC 20611

**Fig. 6:**
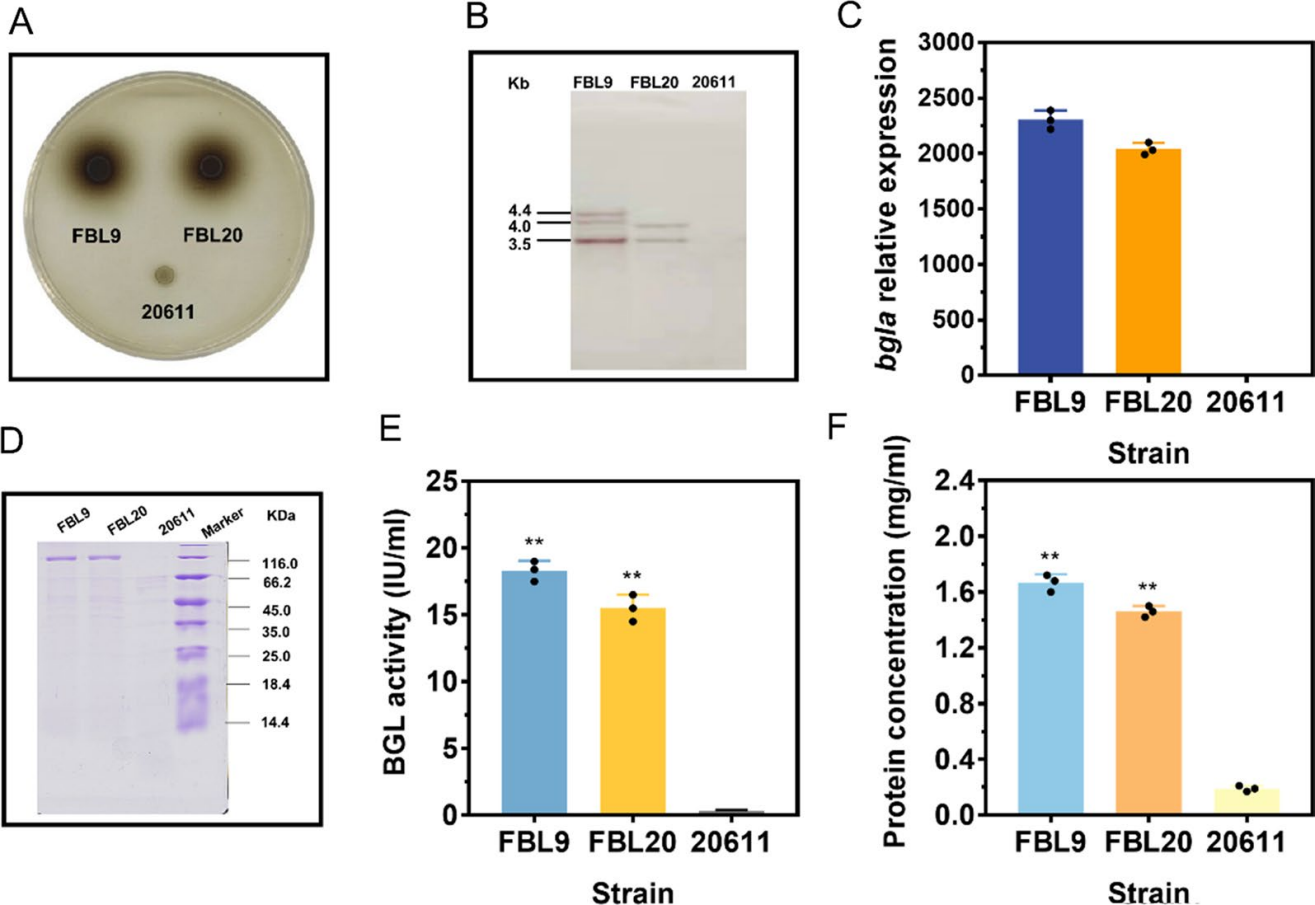
P*fopA*-mediated the β-glucosidase (BGL) gene from *A. niger* C112 (*bgla*) expression in *A. niger*. **A** Detection of BGL activities of *A. niger* strains on the CMC-esculin plate. FBL9 and FBL20 represented recombinant strains expressing *bgla*. 20611 represented the parental strain *A. niger* ATCC 20611. **B** Southern blot assay for confirmation of the *bgla* gene insertion in the genome of *A. niger*. **C** RT-qPCR analysis of the expression levels of *bgla*. *Actin* was used as a reference gene and the 2^−ΔΔCt^ method was used for calculating relative expression levels. **D** SDS-PAGE analysis of the extracellular protein of FBL9, FBL20, and the parental strain ATCC 20611 (20611) on sucrose. **E** BGL activity of FBL9, FBL20, and the parental strain ATCC 20611 cultivated in the sucrose-containing medium after 3 d fermentation. BGL activity was detected using p-Nitrophenyl-β-d-glucopyranoside (pNPG) as the substrate. **F** Quantification of extracellular protein concentrations of FBL9, FBL20, and the parental strain ATCC 20611 (20611) by the Bradford method. Values and standard deviation (SD) of triplicates are presented in the figure. Asterisks indicate statistically significant differences (**p < 0.01) as assessed by Student’s t test. n.d., not detected

Low BGL activity has been recognized as a drawback for efficient cellulose biomass conversion in *Trichoderma reesei* [21, 22]. Thus, supplementing exogenous BGL is a powerful strategy for formulating an efficient cellulolytic cocktail and maximizing saccharification efficiency [42, 43]. In particular, BGLs from *Aspergillus* spp. have higher specific activities and more appropriate kinetic parameters in the hydrolysis of cellobiose in comparison to other fungal BGLs [43–45]. To facilitate access to available BGL for cost-effective biomass conversion, an expression cassette containing the β-glucosidase gene (*bglA*) from *A. niger* C112 under the control of P*fopA* was constructed and co-transformed with pyrithiamine resistance gene (*ptrA*)-containing plasmid pME2892 into *A. niger* ATCC 20611. The candidate transformants were first screened on esculin plates to test the BGL production. The size of the halo around the colony was positively correlated with the BGL activity [20, 21]. Among the transformants, FBL9 and FBL20 showed much larger black halos around the colonies than the parental strain ATCC 20611 (Fig. 6A). Subsequently, the recombinant β-glucosidase (aBGLA) expression strains were further analyzed via Southern blot assay (Fig. 6B). The digested genomic DNA was hybridized by the *bgla* probe, and two fragments (3.5 and 3.8 kb) were found for FBL20 and three bands (3.5, 4.0, and 4.4 kb) were observed for FBL9, while the band specific for the *bgla* gene was not detected in the ATCC 20611 genome. These results suggested that two and three copies of *bgla* were successfully integrated into the genomes of FBL20 and FBL9, respectively.

Furthermore, the two transformants, FBL9 and FBL20, were fermented in FM containing 4% sucrose as the carbon source to investigate their capacity to produce aBGLA. It was found that the relative expression of *bgla* normalized to the *actin* gene exhibited high levels in FBL9 and FBL20, while the transcript of *bgla* was not detected in the parental strain (Fig. 6C). After 72 h of fermentation, the fermentation broths were subjected to the enzyme assay. The secreted proteins from the fungal strains were analyzed by SDS-PAGE without further purification. As shown in Fig. 6D, a clear band of approximately 120 kDa, which was not present in ATCC 20611, was observed in the secreted proteins of FBL9 and FBL20. It was also found that the endogenous protein bands were essentially invisible in the extracellular proteins. MS identification further confirmed that the specific band was the aBGLA expressed by transformants FBL9 and FBL20 (data not shown). These results indicated that the aBGLA was successfully secreted into the culture medium. In accordance with the above result, the BGL activities of FBL9 and FBL20 measured with pNPG as a substrate could reach up to 17.84 U/mL and 15.27 U/mL, respectively (Fig. 6E). Moreover, the extracellular protein concentrations of the fungal strains were also detected. As shown in Fig. 6F, the extracellular protein concentrations of FBL9 and FBL20 (1.68 mg/mL and 1.44 mg/mL, respectively) were significantly increased compared to the parental strain (0.19 mg/mL). It can be estimated that the amount of aBGLA secreted accounted for more than 86.81% of the total amount of extracellular protein. These results revealed that P*fopA* can stimulate high amounts of high-purity BGL production in ATCC 20611 under the sucrose condition.

### Saccharification of the corncob substrates by a combination of the *T. reesei* cellulase mixture and the β-glucosidase produced from *A. niger* FBL9

**Fig. 7:**
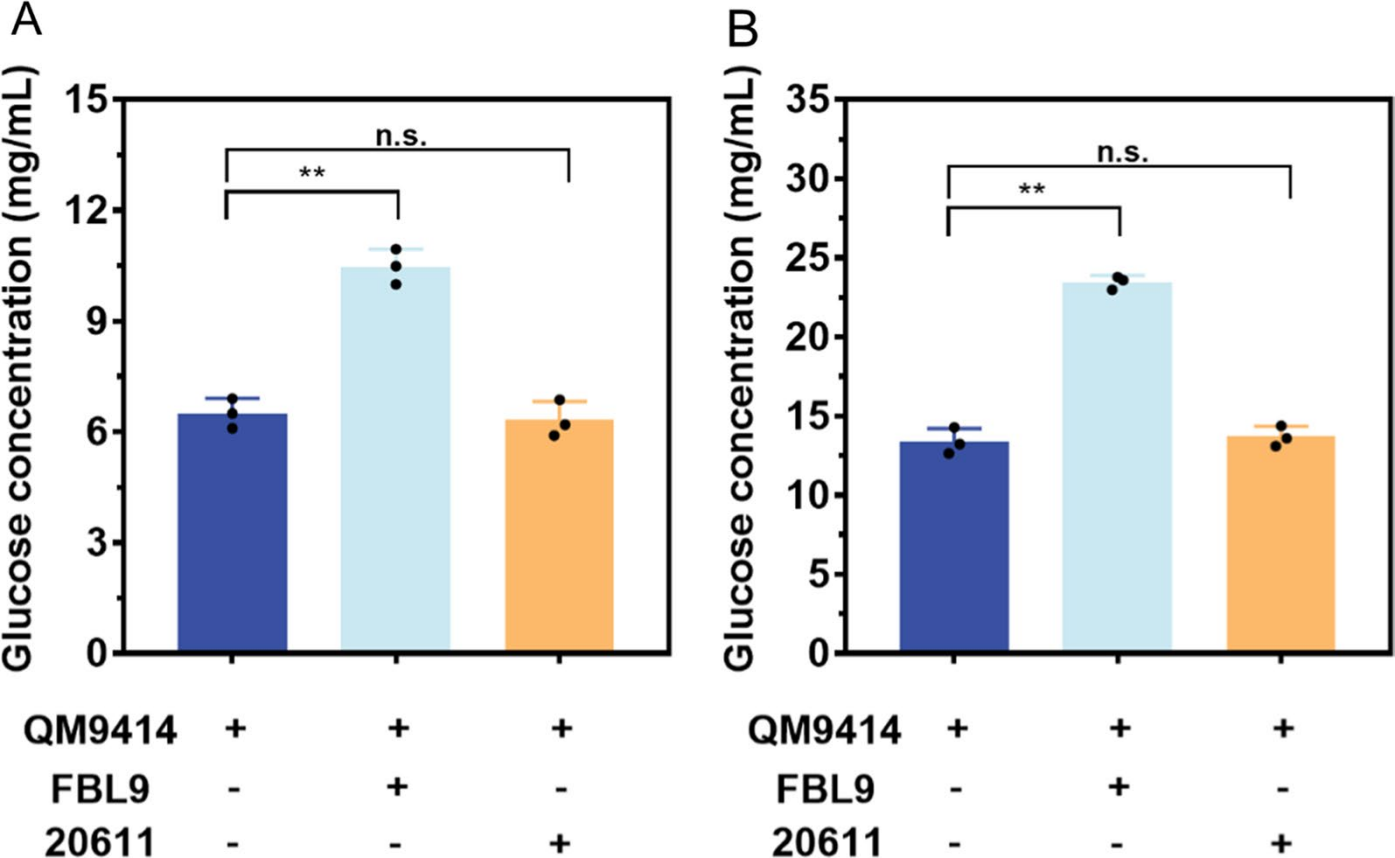
Saccharification of different pretreated corncob residues by the cellulase mixture of *Trichoderma reesei* QM9414 supplemented with the fermentation supernatants of *A. niger* strains. **A** Glucose released from the saccharification of acid-pretreated corncob residue (ACR) by adding the fermentation supernatants of the aBGLA-expression strain FBL9 for 48 h. **B** Glucose released from the saccharification of de-lignified corncob residue (DCR) by adding the fermentation supernatants of the aBGLA-expression strain FBL9 for 48 h. Saccharification of different pretreated corncob residues by adding the fermentation supernatants of ATCC 20611 (20611) was used as the control. Values and standard deviation (SD) of triplicates are presented in the figure. Asterisks indicate statistically significant differences (**p < 0.01) as assessed by Student’s t test. n.s., no significant differences

To test whether the heterologously expressed aBGLA could improve the saccharification ability of the *T. reesei* cellulase mixture, the culture supernatant produced by *A. niger* FBL9, which had the strongest ability to secret BGL among the transformants, was added into the cellulase mixture of *T. reesei* QM9414 with the ratio of the FPA to β-glucosidase activity as 1:1 for saccharification of pretreated corncob residues. As shown in Fig. 7A, when the acid-pretreated corncob residue (ACR) was used as the substrate, the final glucose released by *T. reesei* supplemented with *A. niger* FBL9 after a 48-h reaction was 10.35 mg/mL (corresponding to 29.65% cellulose conversion), which was 56.11% higher than that released by *T. reesei* (6.63 mg/mL, corresponding to 18.99% cellulose conversion). In the saccharification of delignified corncob residues (DCR), the released glucose by *T. reesei* with FBL9 (23.85 mg/mL, corresponding to 68.34% cellulose conversion) was 80.27% higher than the value for *T. reesei* (13.23 mg/mL, corresponding to 37.91% cellulose conversion) after a total enzymatic reaction of 48 h (Fig. 7B). The equal fermentation broth of the parental strain was also added to the *T. reesei* cellulase mixture as a control, but it had no significant effect on saccharification efficiency (Fig. 7A, B). Taken together, the aBGLA produced by *A. niger* FBL9 contributed to optimizing the *T. reesei* cellulase mixture and exhibited superior performance on the saccharification of differently pretreated corncob substrates. Therefore, it has the potential to be a cost-effective complement to traditional cellulase products for application in cellulose bioconversion.

### Sucrose-inducible simultaneous expression of the *T. reesei*-derived chitinase and β-N-acetylglucosaminidase in *A. niger* ATCC 20611

**Fig. 8:**
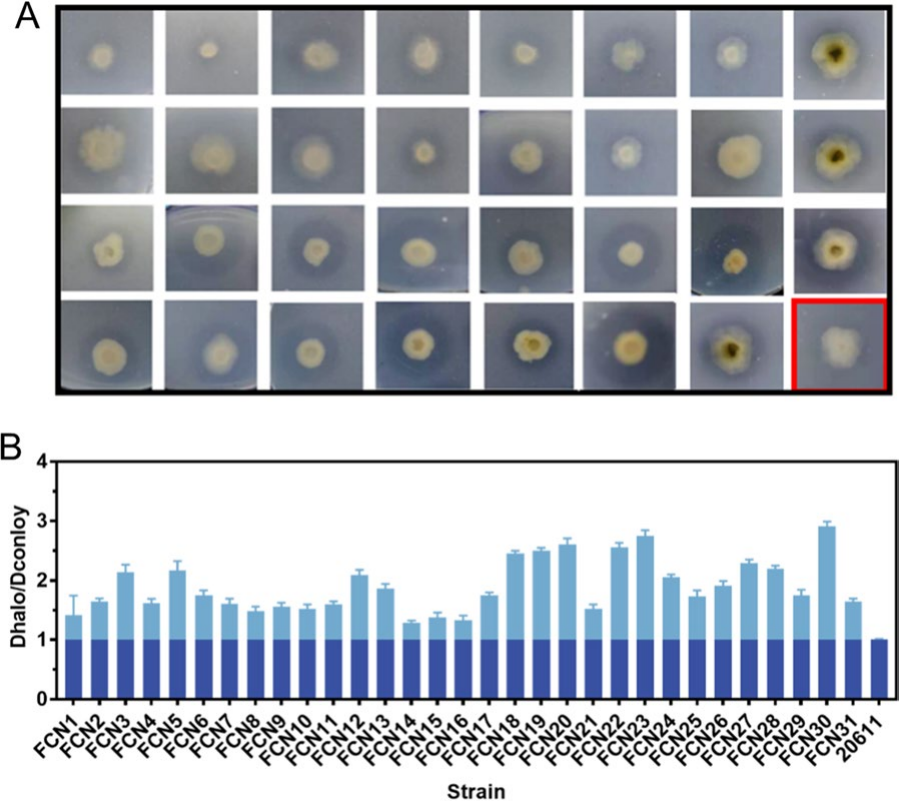
Expression of *T. reesei*-derived chitinolytic enzymes by P*fopA* in *A. niger.* **A** Selection of high chitinolytic enzyme producers by the CD-colloidal chitin plate assay. The *A. niger* strains were grown on the CD agar plate supplemented with 1% colloidal chitin as substrate at 30 ℃ for 72 h. The parental strain *A. niger* ATCC 20611 (20611) was shown as a red box. **B** The ratio of the clear halo diameter to the colony diameter. Values are the average of the triplicates and error bars are the standard deviation (SD) of the triplicates

To date, the heterologous expression of well-performing chitinolytic enzymes at a high level and in a cost-effective manner is essential to optimizing the enzymatic production of GlcNAc [46]. Ike et al. [47] reported that the chitinase Chi46 derived from *T. reesei* could hydrolyze colloidal chitin to yield mainly (GlcNAc)_2_, and Chen et al. [48] found that the GlcNAcase NAG1 derived from *T. reesei* could hydrolyze (GlcNAc)_2_ to produce GlcNAc. To obtain a highly active chitinolytic enzyme-producing strain, the *chi46* and *nag1* expression cassettes under the control of P*fopA* were constructed and co-transformed with the pyrithiamine resistance gene (*ptrA*)-containing plasmid pME2892 into ATCC 20611 to generate transformants. 31 transformants, which grow rapidly on MM plates containing pyrithiamine, were transferred to the CDCC plates to examine their capacity to secrete chitinolytic enzymes. As shown in Fig. 8A, clear chitin hydrolysis haloes were formed around the colonies of transformants harboring the *chi46* and *nag1* expression cassettes, while the parental strain ATCC 20611 could not exhibit a chitin hydrolysis halo. In addition, the hydrolysis halo diameters and the colony diameters of the transformants were measured to indicate the chitinolytic enzyme production. It was found that there were considerable differences in the ratios of halo diameter (D_halo_) to colony diameter (D_colony_) of transformants (Fig. 8B). In particular, a transformant, FCN30, showed the highest value (2.85) in comparison to other fungal colonies, implying its superior ability to produce recombinant chitinolytic enzymes.

**Fig. 9:**
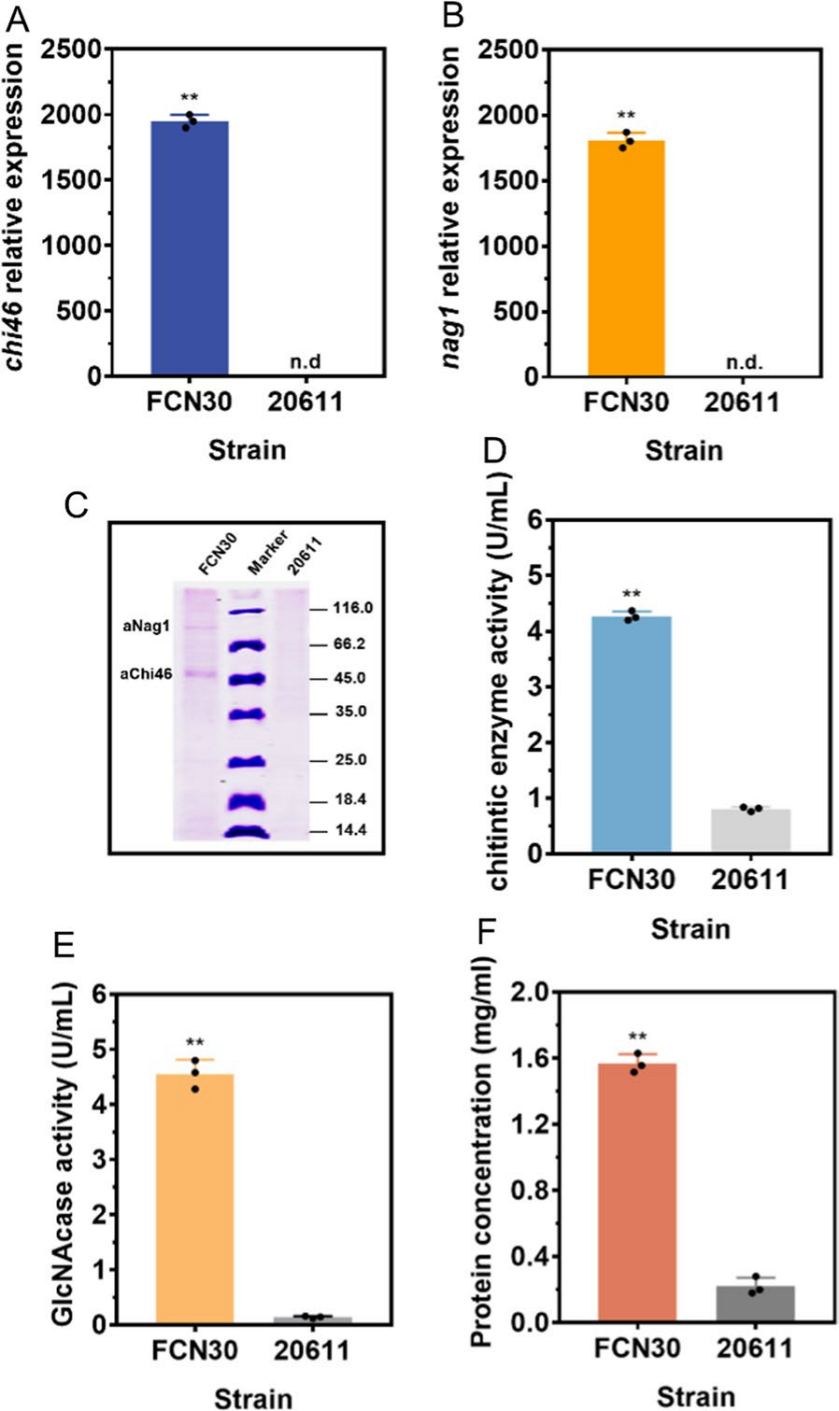
Expression properties of chitinolytic enzymes in *A. niger* FCN30. RT-qPCR analysis of the expression levels of *chi46* (**A**) and *nag1* (**B**) in FCN30. *Actin* was used as a reference gene and the 2^−ΔΔCt^ method was used for calculating relative expression levels. **C** SDS-PAGE analysis of the extracellular protein of FCN30 and the parental strain ATCC 20611 (20611) on sucrose. **D** Determination of the chitinase activities of strains using colloidal chitin as the substrate. **E** Determination of the GlcNAcase activities of strains using pNP-NAG as the substrate. **F** Quantification of extracellular protein concentrations of FCN30 and the parental strain ATCC 20611 (20611) by the Bradford method. Values and standard deviation (SD) of triplicates are presented in the figure. Asterisks indicate statistically significant differences (**p < 0.01) as assessed by Student’s t test. n.d., not detected

Furthermore, FCN30 was fermented using 4% sucrose as a carbon source to determine its capacity to produce chitinolytic enzymes. As shown in Fig. 9A, B, high transcription levels of *chi46* and *nag1* were observed in FCN30, while the transcripts of *chi46* and *nag1* could not be detected in ATCC 20611. The fermentation broth was used for the enzyme assay. First, SDS-PAGE analysis was applied to detect the secreted proteins from the fungal strain. Two specific protein bands at approximately 46 kDa and 80 kDa, which were in accordance with the molecular weights of Chi46 and Nag1, were observed in the fermentation broth of FCN30, while the bands were nonexistent in ATCC 20611. It was also found that the endogenous protein bands were largely invisible in the extracellular protein (Fig. 9C). MS identification further confirmed that the two specific bands were the recombinant chitinase aChi46 and GlcNAcase aNag1 expressed by FCN30 (data not shown). These results showed that the chitinolytic enzymes were successfully secreted into the culture medium. In addition, the activities of chitinolytic enzymes were quantified. When colloidal chitin was used as the substrate, the effect of temperature and pH on enzyme activity was analyzed, and the optimum temperature and pH for the chitinase were found to be 40 °C and pH 5.0 (data not shown). Under this condition, the chitinase activity of FCN30 was 4.26 U/mL, whereas the parental strain exhibited a relatively low enzyme activity of 0.82 U/mL (Fig. 9D). The activity of GlcNAcase in the fermentation broth was also measured using p-Nitrophenyl-N-acetyl–D-glucosamine (pNP-NAG) as the substrate. The optimum temperature and pH for the GlcNAcase were found to be 60 °C and pH 4.0 (data not shown). As shown in Fig. 9E, the GlcNAcase activity of FCN30 could reach up to 4.58 U/mL, while the parental strain possessed a significantly lower enzyme activity of 0.14 U/mL. In addition, the extracellular proteins were quantified by the Bradford method (Fig. 9F). The concentrations of extracellular proteins of FCN30 (1.55 mg/mL) were significantly higher than those of ATCC 20611 (0.20 mg/mL). It could be speculated that the amount of chitinolytic enzymes accounted for more than 87.11% of the total amount of extracellular proteins in FCN30. Taken together, these results revealed that P*fopA* could simultaneously express and secrete the *T. reesei*-derived bienzymatic system of chitinase and GlcNAcase in *A. niger* ATCC 20611 with high purity.

### Bioconversion of colloidal chitin into GlcNAc by the enzyme mixture produced from *A. niger* FCN30

**Fig. 10:**
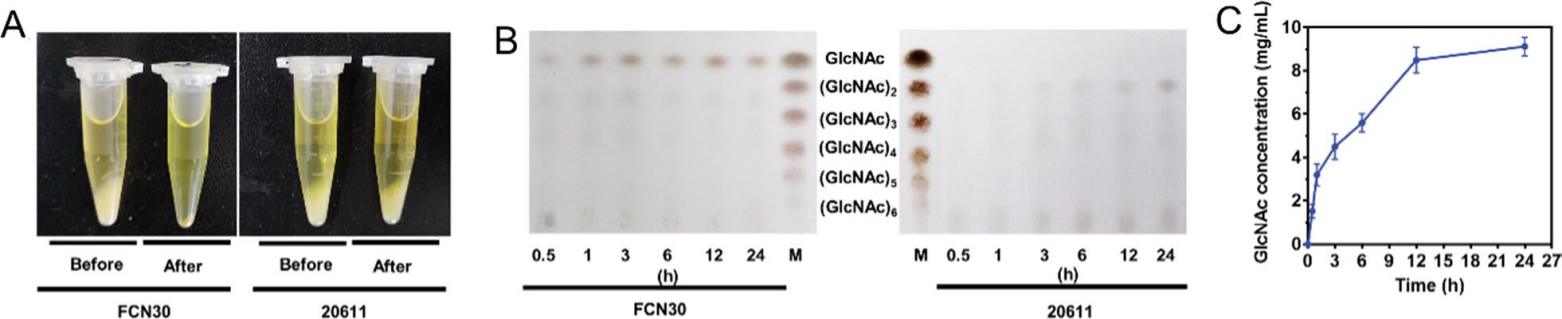
Production of N-Acetyl glucosamine (GlcNAc) by the fermentation supernatant of the chitinolytic enzymes expression strain *A. niger* FCN30. **A** Reaction mixtures containing 1% colloidal chitin as substrate in 50 mM phosphate–citrate buffer (pH 5.0) were incubated with the fermentation broth of FCN30 (left) and ATCC 20611 (right) at 50 ℃ for 24 h. **B** Thin-layer chromatography (TLC) analysis of hydrolysis products of colloidal chitin by FCN30 (left) and ATCC 20611 (right) at different time intervals. Standard N-acetyl-chitooligosaccharides ranging from GlcNAc to (GlcNAc)_6_ were used as the marker. **B** Quantification of GlcNAc produced during the degradation of colloidal chitin by HPLC. Error bars represent the standard deviation (SD) of three replicates. Values are the average of the triplicates and error bars are the standard deviation (SD) of the triplicates

The ability of the chitinolytic enzymes produced by *A. niger* FCN30 to hydrolyze colloidal chitin was investigated. It was found that when the fermentation supernatant of FCN30 was used directly to hydrolyze colloidal chitin, insoluble colloidal chitin was converted to soluble components after 24 h of reaction (Fig. 10A). In contrast, the fermentation supernatant of ATCC 20611 did not significantly reduce the amount of insoluble colloidal chitin (Fig. 10A). Furthermore, the hydrolytic products were analyzed at different time intervals by TLC (Fig. 10B). Following a 30-min incubation, colloidal chitin could be hydrolyzed into GlcNAc, and elongation of the reaction time led to rapid accumulation of GlcNAc. Thus, the chitinolytic enzyme mixture produced by FCN30, which contained aChi46 and aNAG1, efficiently converted colloidal chitin to GlcNAc as the end product. However, the enzyme mixture of ATCC 20611 was unable to hydrolyze colloidal chitin into GlcNAc (Fig. 10B). Subsequently, HPLC was used to quantify the accumulation of GlcNAc in the products of the hydrolysis of colloidal chitin by the chitinolytic enzyme mixture produced by FCN30. At the end of the reaction of 24 h, the highest colloidal chitin conversion ratio of 91.83% at a GlcNAc concentration of 9.18 mg/mL was obtained (Fig. 10C). These results implied that the crude chitinolytic enzyme mixture produced by FCN30 could achieve high-purity GlcNAc production without the assistance of other enzymes and have promising potential for industrial application in chitin valorization.

## Discussion

Over the past few decades, the filamentous fungus *A. niger* has been extensively exploited as a platform organism for the production of heterologous enzymes, benefiting from its outstanding secretory capacity [49, 50]. However, target enzymes expressed in *A. niger* are commonly secreted with endogenous proteins, especially when carbon source-inducible promoters are used, thus affecting enzyme purity [9]. Moreover, carbon source-inducible promoters are commonly regulated by carbon catabolite repression (CCR), and their application may negatively influence target enzyme synthesis as glucose is produced during the growth phase [51]. On the other hand, biomass-degrading enzymes play a vital role in the conversion of biomass to biofuels and bio-products in sugar platform bio-refineries [18, 52]. Nevertheless, cost-effective access to available pure enzymes is considered one of the main obstacles that hinder biomass valorization. This study aimed to develop an efficient sucrose-inducible expression system in *A. niger* ATCC 20611 with minimal endogenous protein contamination for the high-level production of high-purity biomass-degrading enzymes.

Production of target enzymes by *A. niger* expression systems is interfered with by the complex and abundant secretome of the host strains [9, 53]. In particular, the high abundance of hydrolases and proteases in the fungal secretome leads to difficulties in the purification of the target enzymes as well as undesired degradation [4, 5]. Thus, genetic modifications by knocking out the major extracellular proteins, such as glucoamylases, and the proteases, including PepA, PepB, PepD, and PepF, were performed in the *A. niger* natural strains [13, 54, 55]. However, conventional and laborious deletion of multiple genes in fungi is difficult using an inadequate number of transformants and selection markers [12, 40]. Recently, a reasonable strategy of deletion of transcription factors to suppress the downstream extracellular protein expression is adopted to obtain a host with a lower background for enzyme production [9, 40]. For example, the deletion of PrtT, a transcription factor controlling the expression of extracellular protease, significantly enhanced the yield and stability of heterologous proteins [40, 56]. Nevertheless, high-secretory hydrolytic enzymes in the deletion strain still remain, such as alpha-amylase and alpha-glucosidase, leading to a low purity of target enzymes [40, 57]. Interestingly, deletion of AmyR not only reduced the secretion of amylolytic enzymes but also decreased the extracellular protease activity in the *A. niger* ∆*amyR* strain, making *∆amyR* a low protein background host strain [9]. Nevertheless, the absence of AmyR resulted in reduced activity of the commonly used P*glaA* and P*amyA* promoters [9]. Therefore, the expression hosts constructed by the above strategies have not meet the needs of large-scale production of the target enzymes. In this study, the industrial β-fructofuranosidase-producing strain *A. niger* ATCC 20611 was used as the host strain. Unlike commonly used industrial glucoamylase-producing strains, such as CBS513.88 [58], ATCC 20611 grew poorly with starch but efficiently used sucrose to achieve rapid growth and FopA production (Fig. 1). In particular, FopA, the key enzyme responsible for sucrose utilization in ATCC 20611, was basically not secreted into the fermentation medium (Fig. 2). Furthermore, the extracellular protease activity of the strain under sucrose culture conditions was very low, with only 16.59% of *A. niger* MGG029, a *prtT*-deletion strain (Fig. 2, Additional file 1: Fig. S1). Thus, the extracellular protein background of *A. niger* ATCC 20611 was relatively clean, and its endogenous protein interfered minimally with the production of the target enzyme. Compared to other *A. niger* hosts [9, 13, 40], *A. niger* ATCC 20611 is an excellent host for enzyme production without disrupting the internal regulatory circuits.

The efficient expression of target enzymes depends largely on a robust promoter [3, 49, 51]. Generally, the application of inducible promoters from glycoside hydrolases, such as glucoamylase, xylanase, and amylase, is an overwhelming strategy to achieve high yields of heterologous enzyme production [59]. However, these promoters remain subject to several limitations that hinder their widespread application, such as leakage under non-inductive conditions, carbon catabolite repression, and endogenous protein interference [9, 49, 50]. This study identified a novel sucrose-inducible promoter of the β-fructofuranosidase gene, P*fopA*, which was strongly induced only under sucrose conditions and completely repressed under non-inducible conditions. In particular, the existence of a positive correlation between gene relative expression and sucrose concentration made this promoter a promising candidate for fine-tuning gene expression. Moreover, P*fopA* could be activated by sucrose even in the presence of glucose, indicating the activity of this promoter was not repressed by glucose (Fig. 4). This property allows *A. niger* ATCC 20611 to use agro-industrial wastes, such as sugarcane bagasse (containing sucrose, glucose, and fructose), as a carbon source instead of pure sucrose for growth and enzyme production, enhancing the economics of applying this promoter. In addition, since FopA was essentially not secreted into the medium, P*fopA* was enabled to express the target enzyme without interference from endogenous proteins, resulting in highly pure enzyme production (Fig. 3). To our knowledge, some sucrose-inducible promoters have already been reported in bacteria, yeasts, and other *A. niger* strains for intra- and extracellular protein production [50, 60, 61], such as the promoter of a levansucrase (P*sacB*) from *Bacillus megaterium* [60]. However, given that SacB is a secreted protein, the use of P*sacB* results in the background protein being secreted along with the target enzyme, thus reducing the purity of the target enzyme. And P*sacB* has basal promoter activity under non-inducing conditions, which is detrimental to the expression of somehow unfavorable or deleterious for the host. As for the promoter of invertase (P*suc2*) from *Saccharomyces cerevisiae* [61], the P*suc2*-regulated genes are tightly repressed by glucose, and thus the P*suc2*-driven protein expression requires the use of purified sucrose as a substrate, which is uneconomical for large-scale cultivation of industrial fungi. Especially, a series of expression vectors based on the P*sucA* promoter have been constructed in *A. niger* ARAn10 for the intracellular production of recombinant proteins [50]. However, time-consuming procedures about cell disruption, protein extraction, and affinity chromatography purification are required to obtain high-purity recombinant proteins. In contrast to these sucrose-inducible promoters, P*fopA* has the advantages of low background protein secretion, high efficiency, tight control, and not being subject to catabolite repression, making it a promising genetic tool for implementing dynamic regulatory systems to express enzymes in *A. niger*.

The economic utilization of cellulose or chitin as the feedstock in the bio-refinery industry represents a profound shift in industrial carbon utilization, in which the biopolymer must first be broken down into constituent monosaccharides for further bioconversions [62]. Hence, there is a rising demand for biomass-degrading enzymes, but cost remains a limiting factor for the widespread use of enzymes. Especially for the acquisition of pure enzymes, common protocols for purifying enzymes usually include fractional precipitation, ion exchange, or gel permeation chromatography, which will increase the cost of enzyme production [31–33, 40]. In this regard, the application of expression systems with a low background protein secretion would facilitate the easy purification of the target enzyme or, in some cases, even eliminate the need for additional purification steps. In this study, the sucrose-inducible expression system was successfully utilized for the production of a BGL from *A. niger* C112, which is the rate and cost-limiting enzyme for efficient utilization of cellulose. The BGL activity of FBL9 could reach 17.84 U/mL, which is higher than the previous report, in which the BGL yield ranged from 5.87 to 8.91 U/mL in different expression systems [63, 64]. Notably, the enzyme samples could be obtained directly from the supernatant after 72 h of cultivation without any purification procedure, and the purity of the secreted aBGLA was estimated to be over 86% (Fig. 6D, E). The ability of a BGL in the synergetic enzymatic saccharification process is undoubtedly a symbolic index for estimating its utility value [24]. The high purity of aBGLA provided an important prerequisite for the direct application of the fermentation supernatant to degraded corncob residues. When the fermentation supernatant was added to the *T. reesei* cellulase mixture and the ratio of FPA to BGL was increased to 1:1, the saccharifying ability toward ACR and DCR improved by 56.11%, and 80.27%, respectively. In comparison, when the ratio of FPA and purified pBGL1 from *Penicillium decumbens* C114-2 was 1:1, the saccharifying ability toward ACR and DCR improved only 41.9% and 65.5% respectively. When the ratio of FPA and BGL was 1:1 by adding the commercial enzyme Novozyme NS-50010, the saccharifying ability toward ACR and DCR enhanced by 60.5% and 87.4% respectively. [42]. Thus, aBGLA seems to be more efficient and cost-effective in boosting the saccharification process than pBGL1. aBGLA is not as efficient as Novozyme NS-50010, probably because Novozyme NS-50010 preparation contains other (hemi)cellulase in addition to BGL, which will facilitate the conversion of the substrate [42]. Collectively, the successful production and application of aBGLA not only provide a favorable supplement to reduce the cost of industrial cellulose degradation but also demonstrate that this expression system could be expected to produce other bulk enzymes.

At present, studies on the synergistic action of chitinase and GlcNAcase to hydrolyze colloidal chitin for GlcNAc production have been reported [31–33]. In this study, a recombinant *A. niger* strain FCN30 co-expressing the *T. reesei* chitinase Chi46 and GlcNAcase NAG1 was constructed using the sucrose-inducible expression system, and the fermentation broth of this strain was further used to convert colloidal chitin. It was found that the enzyme cocktail of FCN30 efficiently hydrolyzed colloidal chitin and generated GlcNAc as the only end product, yielding 9.18 mg/mL with a high conversion ratio of up to 91.83% after 24-h incubation (Fig. 9). The final conversion ratio is much higher than those of previous reports (62.2% [65]; 79% [66]; 80.2% [31]; 83% [28]; 85% [67]), and is near to that of the report by Fu et al. (92.6% [32]) and Cardozo et al. (93% [68]). It is noteworthy that the reaction time (24 h) in this study is much shorter than those of previous reports (2 d [69, 70]; 5 d [28]; 7 d [67]; 10 d [71]). A further advantage is that the enzyme cocktail of FCN30 is purification-free and can be used directly for chitin degradation to achieve the same conversion rate as using heterologously expressed and purified chitinolytic enzymes. Thus, the recombinant *A. niger* strain FCN30 could be a good candidate for GlcNAc production. Furthermore, the successful construction and application of FCN30 demonstrate the potential of this sucrose induction system for the simultaneous expression of multiple genes, providing a reference for the development of *A. niger* ATCC 20611 as a cell factory.

In general, promoter strength can be further enhanced by genetic modification, including deleting repressor binding sequences and adding activator binding sequences [12]. As a consequence, the identification of transcription factors that regulate *fopA* expression is a prerequisite for modifying the P*fopA*. As a reference, a Zn(II)2Cys6-type positive-acting transcription factor InuR has been identified in N402, which is essential for the induced expression of genes involved in inulin and sucrose metabolism [72]. However, the InuR binding sequence provided in their study has not been experimentally confirmed. Future studies are needed to focus on the transcription factors that regulate *fopA* expression and their binding sequences, which will be meaningful for the construction of artificial promoters to further improve enzyme production at the promoter level. In addition, sucrose molasses is considered to be a more promising inducer for bio-refineries than pure sucrose because of its higher cost-effectiveness and rich sugar content [73]. The economic efficiency of industrial enzymes can be further improved by replacing the carbon source of the fermentation medium.

## Conclusions

In this study, a sucrose-inducible expression system was developed based on an adjustable promoter, P*fopA*, and a low background host strain, *A. niger* ATCC 20611. The system was successfully used to produce the mono-enzyme β-glucosidase and the chitin-degrading enzyme cocktail consisting of chitinase Chi46 and GlcNAcase NAG1 at high levels. The fermentation broths of recombinant strains can be directly applied to the substrate without additional purification procedures, enabling efficient and cost-effective biomass degradation. This study not only facilitates the implementation of the industrial strain *A. niger* ATCC 20611 as an efficient enzyme-producing cell factory but also provides a purification-free expression platform for bulk enzyme production.

## Materials and methods

### Strains and culture media

*A. niger* ATCC 20611, an important industrial fructo-oligosaccharides-producing strain, was used as the parental strain for *A. niger* transformation. *A. niger* MGG029 ((*prtT, glaA∷fleo*^*r*^*pyrG*) [74] was used as a control strain for the protease production assay. The cellulase producer *T. reesei* QM9414 was used for the saccharification of pretreated corncob residues [21]. Strains were grown on the potato dextrose agar plate (PDA) for conidia production [35]. The spore suspension was obtained by washing the PDA plate with solution A (containing 0.9% NaCl and 0.5% Tween 80). Fermentation medium (FM) was used for *A. niger* cultivation, FopA production, and sugar consumption assay [35]. Czapek-Dox medium (CD) was used for *A. niger* cultivation and sugar consumption assay [35]. *T. reesei* QM9414 was inoculated as described by Qian et al. for cellulase production [21]. Transformation medium was used for protoplast regeneration [36]. A minimal medium (MM; [75]) supplemented with 2 μg/mL pyrithiamine (Sigma, USA) was applied for assessing the genetic stability of transformants. And the MM agar plate containing sucrose (2%) or glucose (2%) as carbon resources was used to observe the fluorescence of the *A. niger* strains. The skim milk-agar plate was used to determine the protease secretion ability of strains [39]. The esculin plate was used to screen the transformants with high β-glucosidase activity [21]. The CD agar plate containing colloidal chitin was applied for screening of high chitinolytic enzyme producers.

### Fungal growth, β-fructofuranosidase production, extracellular protease production, and sugar consumption

The spore suspension (10^8^ spores/mL) of *A. niger* ATCC 20611 was inoculated in 200 mL of FM/CD. Following the sampling of these cultures, the mycelia were separated from the culture broth to measure fungal biomass and β-fructofuranosidase activity and the culture broth was used to analyze extracellular protease production and sugar consumption during the fermentation process.

After 10 mL of the cultures were filtered through a Buchner funnel, the collected mycelia were washed with distilled water three times and then dried at 85ºC to constant weight. The dry weight of mycelia was calculated as the difference between the weights of the dried filter paper with and without mycelia.

β-fructofuranosidase activity was determined according to the method described by Zhang et al. [36]. One unit (U) was defined as the amount of enzyme required to produce 1 μmol of reducing sugars per min. Protease activity was detected according to the method described by Sun et al. [39] with protease K (Genview, USA) as the standard.

SBA-40C biological sensor analyzer (BISAS, Shandong, China) was used to detect the glucose concentration at different time points during the fermentation process. Sucrose Content Assay Kit (Solarbio Science & Technology Co., Ltd., Beijing, China) was used to detect the sucrose concentration at different time points during the fermentation process.

### Expression cassette and deletion cassette construction

Phanta^®^ Super-Fidelity DNA Polymerase (Vazyme Biotech Co., Ltd., Nanjing, China) was used for PCR amplification. Primers were designed using the primer premier 5.0 software and were listed in Additional file 2: Table S1.

A 2.2-kb fragment located upstream of the FopA-coding sequence (*fopA*) from the *A. niger* ATCC 20611 genome was amplified using primer pair P*fopA*-UF1/ P*fopA*-UR1 and used as the P*fopA* promoter. The fragments of P*gpdA* and T*trpC* were amplified from plasmid pAN7-1 (GenBank accession number Z32698.1) with prime pairs P*gpdA*-UF/P*gpdA*-UR and T*trpC*-F/T*trpC*-R, respectively. The genomic DNA of *T. reesei* QM6a (GenBank accession number GCA_000167675.2) was used as the template to amplify the terminator T*cbh1* using primer pair T*cbh1*-1DF*/*T*cbh1*-1690DR.

For construction of an enhanced green fluorescence protein (EGFP) expression cassette, Plasmid pIG1783 [36] was used as the template to amplify the EGFP encoding gene *egfp* with the primer pair egfp-F (P*fopA*)/egfp-R (T*trpC*). P*fopA*/P*gpdA*, *egfp*, and T*trpC* were fused by Double-joint PCR to get the final EGFP_P*fopA*_ and EGFP_P*gpdA*_ expression cassette using the primer pair Pf*opA*-UF1/ T*trpC*-R and P*gpdA*-UF1/ T*trpC*-R.

For construction of a β-glucosidase (BGLA) expression cassette, the BGLA coding sequence was cloned from *A. niger* C112 genome (GenBank accession number KP307454.1) using the primer pair bgla-F (P*fopA*)/bgla-R (T*trpC*). The *bgla* fragment was fused with P*fopA* and T*trpC* to get the BGLA expression cassette using the primer pair P*fopA*-UF1/ T*trpC*-R.

For construction of a chitinase (Chi46) expression cassette, the Chi46 coding gene (GenBank accession number GCA_000167675.2) was amplified from the genomic DNA of *T. reesei* QM6a using the specific primers chi46-F (P*fopA*)/chi46-R (T*cbh1*). Then the *chi46* fragment was fused with P*fopA* and T*cbh1* to get the expression cassette using the primer pair P*fopA*-UF2/ T*cbh1*-R.

For construction of a β-N-acetylglucosaminidase (Nag1) expression cassette, the signal peptide of the cellobiohydrolase I gene (*cbh1*) from *Trichoderma reesei* QM6a (GenBank accession number GCA_000167675.2) was synthesized and added to the 5ʹ end of the *nag1* gene using the primer pair nag1-F (SP*cbh1*)/nag1-R (T*trpC*). Then, the *nag1* fragment was fused with P*fopA* and T*trpC* to get the NAG1 expression cassette using primer pair P*fopA*-UF2/ T*trpC*-R1.

To generate the *fopA* deletion cassette, the 5ʹ and 3ʹ flank fragments of *fopA* were amplified from the genome of *A. niger* ATCC 20611 using the prime pairs P*fopA*-UF1/ P*fopA*-UR1 and T*fopA*-F/T*fopA*-R, respectively. The expression cassette of resistance gene *ptrA* was amplified from the plasmid of pIG1783 using the prime pair ptrA-F (P*fopA*)/ptrA-R (T*fopA*). Then the three purified DNA fragments were fused to amplify the FopA deletion cassette using the prime pair P*fopA*-UF1/ T*fopA*-R1.

For *fopA* overexpression cassette construction, the *fopA* gene from the *A. niger* ATCC 20611 genome was amplified using primer pair fopA-F (P*gpdA*)/fopA-R (T*trpC*). Then, the *fopA* fragment was fused with P*gpdA* and T*trpC* to get the *fopA* overexpression cassette with the primer pair P*gpdA*-UF/T*trpC*-R1 using Double-joint PCR method [76].

### Transformation of *A. niger* ATCC 20611

The polyethylene glycol (PEG)/CaCl_2_-mediated transformation of protoplasts was performed based on the method described by Zhang et al. [36]. Protoplasts of *A. niger* ATCC 20611 were co-transformed with the expression cassette and the plasmid pME2892 [36], which carries the selectable marker *ptrA* gene. All target transformants were purified three times via single-colony isolation on the MM plate containing 2 μg/mL Pyridinium thiamine (Sigma, USA).

### Expression of EGFP in *A. niger*

For detection of EGFP expression, the spores of the *A. niger* strain were spotted on MM-sucrose/MM-glucose plates with coverslips and grown for 48 h at 30 °C. EGFP expressed in mycelia was visualized using fluorescence and light microscopy (Nikon Eclipse 80i fluorescence microscope).

For quantification of the fluorescence intensity, the *A. niger* strains were grown in FM for 48 h. Mycelial cells were subjected to fluorescent intensity analyses using a multimode plate reader (Tecan Infinite Pro 200, Switzerland) with excitation and emission wavelengths of 488 and 520 nm, respectively. The parental strain of ATCC 20611 without an integrated EGFP expression cassette was regarded as a control.

## Enzyme assay

### β-Glucosidase activity assay

For visualization of BGL expression and secretion, the purified transformants containing BGLA expression cassette were grown onto CMC-esculin plates at 30℃ for 10 h. To further quantify the expression of β-glucosidase, the spores of transformants and the parental strain ATCC 20611 were cultured in FM with 4% sucrose as a carbon source at 30 ℃, 200 rpm for 72 h. The fermentation supernatants were collected by centrifugation at 10,000 rpm for 10 min and filtered by a 0.22-micron membrane. The β-glucosidase activity was measured using p-Nitrophenyl-β-d-glucopyranoside (pNPG) as a substrate [21]. One unit of enzyme activity was defined as the amount of enzyme required that liberated 1 μmol p-nitrophenol per min under the assay condition, with p-nitrophenol as the standard.

### Chitinolytic enzyme activity assay

The ability of the strain to produce chitinolytic enzymes was first assessed using colloidal chitin as a substrate. Colloidal chitin was prepared as described by Sandhya et al. [77]. *A. niger strains* were plated on CD-colloidal chitin (1%) agar plates (CDCC) and grown for 72 h. To further determine the production of chitinolytic enzymes, the spores (10^6^/mL) of transformants FCN30 and the parental strain ATCC 20611 were cultured in FM with 4% sucrose as a carbon source at 30 °C and 200 rpm for 96 h. The fermentation supernatants were collected by a 0.22-micron membrane and subjected to enzyme assays.

The activity of chitinolytic enzymes was determined following the method of Sandhya et al. [77] using colloidal chitin as a substrate. One unit (U) of chitinase activity was defined as the amount of enzyme required to liberate 1 μmol of reducing sugars per minute under the detection condition.

p-Nitrophenyl-N-acetyl-β-d-glucosaminide (pNP-NAG) was used as the substrate to determine the GlcNAcase activity [78]. One unit of enzyme activity was defined as the amount of enzyme that produced 1 μmol of p-nitrophenol per minute under the assay conditions.

### Extracellular protein assay

The protein concentration of the fermentation broth was measured using the Bio-Rad DC Protein Assay kit (Sangon Biotech, Shanghai, China) with bovine serum albumin (BSA) as the standard protein.

For the SDS-PAGE assay, samples were prepared by boiling fermentation supernatants (10 μL) with loading buffer (containing β-Mercaptoethanol) at 100 °C for 10 min. The Unstained Protein Molecular Weight Marker (ThermoScientific, Carlsbad, CA, USA), ranging from 14.4 kDa to 116.0 kDa, was used as the size standard. The protein electrophoresis was performed with a 5% stacking gel and a 12% separating gel, and then the protein bands were visualized with Coomassie Brilliant Blue R-250 staining and acetic-acid/methanol/water (2:3:35, v/v/v) decolorizing. The predicted bands were excised for MALDI-TOF-MS identification.

### Saccharification assay of pretreated corncob residues

ACR and DCR were used as substrates in the saccharification process [22]. *T. reesei* QM9414 was cultured as described by Qian et al. [21]. The fermentation broth was collected by centrifugation at 10,000 rpm for 5 min and subjected to filter paper activity (FPA) and BGL activity assays [21, 22]. 5% corncob residue was used as substrate. The enzyme mixture with equal FPA activity (10 U/g substrates) and BGL activity (10 U/g substrates) was loaded by adding citric acid buffer (pH 4.8) to make up the total volume to 30 mL. The reaction was carried out at 50 °C for 48 h, and the amount of glucose released was measured with an SBA-40C biological sensor analyzer (BISAS, Shandong, China). Cellulose conversion was calculated as described by Qian et al. [21].

### Conversion of colloidal chitin to GlcNAc

The degradation efficiency of chitinolytic enzymes from *A. niger* was analyzed using TLC with colloidal chitin as the substrate. The reaction mixture containing 1% (w/v) colloidal chitin prepared in 50 mM citrate buffer (pH 5.0) with 1 mL of the fermentation broth was incubated at 50 °C. Aliquots were withdrawn at various time intervals, and the reaction was immediately stopped by boiling for 10 min. After centrifugation (10,000 g, 10 min), the supernatants were spotted onto a TLC Aluminum Silica Gel 60 F254 plate (Merck, Germany). TLC plates were developed with n-butanol/methanol/ammonia/water (5:4:2:1, v/v/v/v) and sprayed with aniline-diphenylamine (DPA) reagent, followed by heating at 105 °C in an oven until spots were visualized. N-acetyl-chitooligosaccharides ranging from GlcNAc to (GlcNAc)6 were used as standards.

The concentration of GlcNAc was quantitatively analyzed by HPLC, using a Shimadzu-LC-20A series instrument (Kyoto, Japan) equipped with a RID-20A detector and a Zorbax carbohydrate column (250 × 4.6 mm, 5 μm, Agilent Technologies). The column temperature was maintained at 45 °C. The isocratic elution condition for N-acetylglucosamine (GlcNAc) was set to acetonitrile/water (80:20, v/v) at a flow rate of 1 mL/min. GlcNAc was used as a standard. The GlcNAc concentration of the reaction was calculated by comparing the peak area to the standard solution. The conversion rate is the percentage of released GlcNAc weight (mg) to initial colloidal chitin weight (mg) [31].

### Total RNA extraction and reverse transcription quantitative real-time PCR (RT-qPCR)

For total RNA extraction, approximately 200 mg of ATCC 20611 mycelia was harvested by a filter, then the wet mycelia were frozen by liquid nitrogen immediately. Total RNA was extracted using Trizol Reagent, according to the manufacturer’s protocol. PrimeScript^™^ RT reagent kit with gDNA Eraser (TaKaRa, Dalian, Liaoning, China) was used to synthesize cDNA. Primers for RT-qPCR were listed in Additional file 2: Table S1. The SYBR Premix and 1 μl of appropriately diluted cDNA were used with 200 nM of forward and reverse primers in a final volume of 10 μl. RT-qPCR protocols were set following the instructions from the SYBR Premix Ex Taq^™^ (Tli RNaseH Plus) kit (TaKaRa, Dalian, Liaoning, China) manufacturer*.* Gene transcription level was analyzed using LightCycler 480 System. *Actin* was used as the reference gene and the 2^−ΔΔCt^ method was applied for calculating relative expression levels.

### Southern blot analysis

The probe of *bgla* was a fragment amplified through PCR using primer bgla-430F/bgla-1206R to detect the *bgla* gene. The BgIII/BamHI-digested genomic DNA was hybridized by the *bgla* probe. The probe-hybridized DNA fragment was detected with the DIG High Prime DNA Labeling and Detection Starter Kit I according to the manufacturer’s protocol (Roche Diagnostics, Mannheim, Germany).

### Statistical analysis

Student’s t test was used to analyze the significant difference. Significant differences were marked at *p* < 0.05 and *p* < 0.01 levels.

## Supplementary Information

**Additional file 1: Figure S1.** Detection of extracellular protease production by *A. niger* ATCC 20611 and *A. niger* MGG029. (A) Skim milk-agar plate assay for protease activity. Colonies of ATCC 20611 (left) and MGG029 (right) were grown on skim milk-agar plates for 3 d. (B) The ratios of halo diameter (D_halo_) to colony diameter (D_colony_). (C) Detection of protease activity in the supernatant of ATCC 20611 and MGG029 using azoic casein as substrate. Values and error bars of triplicates are presented. Asterisks indicate statistically significant differences (**p < 0.01) as assessed by Student’s t test.

**Additional file 2: Table S1.** Primers used in the present study.

## Data Availability

The data supporting the conclusions of this article are included in this article and its Additional files. Further datasets used and analyzed during the current study are available from the corresponding author upon reasonable request.

## Abbreviations

FopA: β-Fructofuranosidase
FOS: Fructooligosaccharides
BGL: β-Glucosidase
GlcNAcase: β-N-acetylglucosaminidase
GlcNAc: N-acetyl-D-glucosamine
pNPG: P-Nitrophenyl-β-d-glucopyranoside
pNP-NAG: P-Nitrophenyl-N-acetyl–β-d-glucosamine
FM: Fermentation medium
CD: Czapek-Dox medium
MM: Minimal medium
FPA: Filter paper activity
ACR: Acid-pretreated corncob residues
DCR: Delignified corncob residues
