# Supplementary figures and images for: A novel sucrose-inducible expression system and its application for production of biomass-degrading enzymes in Aspergillus niger
Source: Biotechnol Biofuels Bioprod. 2023 Feb 13;16:23. doi: 10.1186/s13068-023-02274-7 (PMC9926565; doi:10.1186/s13068-023-02274-7)

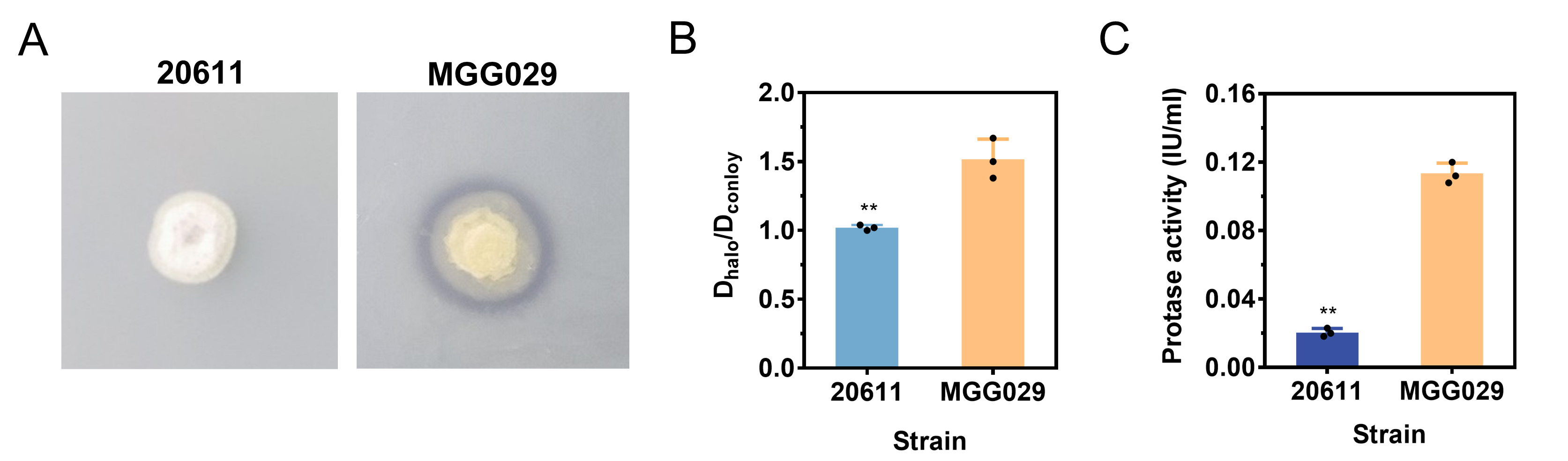

Supplement: Supplementary file 1 — Additional file 1: Figure S1. Detection of extracellular protease production by A. niger ATCC 20611 and A. niger MGG029. (A) Skim milk-agar plate assay for protease activity. Colonies of ATCC 20611 (left) and MGG029 (right) were grown on skim milk-agar plates for 3 d. (B) The ratios of halo diameter (Dhalo) to colony diameter (Dcolony). (C) Detection of protease activity in the supernatant of ATCC 20611 and MGG029 using azoic casein as substrate. Values and error bars of triplicates are presented. Asterisks indicate statistically significant differences (**p < 0.01) as assessed by Student’s t test. [file 13068_2023_2274_MOESM1_ESM.tif]
