# Supplementary material for: A novel sucrose-inducible expression system and its application for production of biomass-degrading enzymes in Aspergillus niger
Source: Biotechnol Biofuels Bioprod. 2023 Feb 13;16:23. doi: 10.1186/s13068-023-02274-7 (PMC9926565; doi:10.1186/s13068-023-02274-7)
Supplement: Supplementary file 2 — Additional file 2: Table S1. Primers used in the present study. [file 13068_2023_2274_MOESM2_ESM.docx]

Table S1 Primers used in this study

| **Primer name** | **Sequences (5′–3′)** | |
| --- | --- | --- |
| **Primers for gene amplification, expression cassette construction, and deletion cassette construction** | |  |
| P*fopA*-UF1 | AGGGGAATTACGATGTTTGC | |
| P*fopA*-UF2 | ACTGGTCACTCTTTTGGCA | |
| P*fopA*-UR1 | CATTGTGATGCTCCTGGATTG | |
| egfp-F (P*fopA*) | CAATCCAGGAGCATCACAATGGTGAGCAAGGGCGAGGAGC | |
| egfp-R (T*trpC*) | TTTGATGATTTCAGTAACGTTAAGTTGGACGAGCTGTACAAGTAA | |
| bgla-F (P*fopA*) | CAATCCAGGAGCATCACAATGAGGTTCACTTTGATCGAG | |
| bgla-R (T*trpC*) | TTTGATGATTTCAGTAACGTTAAGTTTAGTGAACAGTAGGCAGAGACG | |
| P*fopA*-UR1 (SP*cbh1*) | AGCACGAGCTGTGGCCAAGAAGGCCGAGATGACGGCCAACTTCCGATACATTGTGATGCTCCTGGATTG | |
| nag1-F (SP*cbh1*) | ATGTATCGGAAGTTGGCCGTCATCTCGGCCTTCTTGGCCACAGCTCGTGCTATGCTGCTGCCCAAGGCCGTCCT | |
| nag1-R (T*trpC*) | TTTGATGATTTCAGTAACGTTAAGTTCATGGGAAAAGGGCGCAGGCCGTC | |
| chi46-F (P*fopA*) | CAATCCAGGAGCATCACAATGTTGAACTTCTTCGG | |
| chi46-R (T*cbh1*) | GGCTTTCGCCACGGAGCTCACAGATGCCGAATACGAGG | |
| T*cbh1*-1DF | AGCTCCGTGGCGAAAGCC | |
| T*cbh1*-1690DR | GAATCACAAACCGCCAAAGC | |
| T*trpC*-F | ACTTAACGTTACTGAAATCATCAAA | |
| T*trpC*-R | GAGTGGAGATGTGGAGTGGG | |
| T*trpC*-R1 | TTCTGGGTAAACGACTCATAG | |
| ptrA-F (P*fopA*) | CAATCCAGGAGCATCACAATG AGGGTAAGGTCGTGGTTT | |
| ptrA-R (T*fopA*) | ATAGCGAGGAGTTACCAATGGA TCCTCAACAGCGGTAGCAT | |
| T*fopA*-F | TCCATTGGTAACTCCTCGCTAT | |
| T*fopA*-R | GGAATCAAGATGTTGGGACGA | |
| T*fopA*-R1 | GGTTGTTCCACTTTGAGGCA | |
| P*gpdA*-UF | AGACCTAATACAGCCCCTAC | |
| P*gpdA*-UR | TGTCTGCTCAAGCGGGGTAG | |
| fopA-F (P*gpdA*) | CTACCCCGCTTGAGCAGACA ATGAAGCTCACCACTACCACCC | |
| fopA-R (T*trpC*) | TTTGATGATTTCAGTAACGTTAAGT TCAATTTCTCTCCGGCCAG | |
| **Primers for RT-qPCR** | |  |
| qactin-F | TGGTATCTCCGACCGTATGC | |
| qactin-R | AAGCACTTGCGGTGGACG | |
| qgpdA-F | CTACGACGAGATCAAGCAGACC | |
| qgpdA-R | CTTGACGAAGTTGGGGTTG | |
| qfopA-F  qfopA-R | TCTCACTGTCGTCGTGGATAA  TCAATTTCTCTCCGGCCAG | |
| qegfp-471F | GCAGAAGAACGGCATCAA | |
| qegfp-667R | CCAGCAGGACCATGTGAT | |
| qbgla-2732F | AGATCGTGCTGCGTCAA | |
| qbgla-2844R | GTCCTGCTTCTCAACATTCC | |
| qchi46-1120F | GACCGTCCAGTACGATGA | |
| chi46-1320R | CTTGTGGCTTGTTCCAAT | |
| qnag1-1450F | TCTGGAGCGAGATGATTGACG | |
| qnag1-1645R | CTGGATGGGCATAGCACTGAC | |
| **Primers for Southern blot** | |  |
| bgla-430F | GGCGACAGATTGGGAGTT | |
| bgla-1206R | GAGCACGCTAATGGTCAAG | |
